# Identification of Potential Bisphenol A (BPA) Exposure Biomarkers in Ovarian Cancer

**DOI:** 10.3390/jcm10091979

**Published:** 2021-05-05

**Authors:** Aeman Zahra, Qiduo Dong, Marcia Hall, Jeyarooban Jeyaneethi, Elisabete Silva, Emmanouil Karteris, Cristina Sisu

**Affiliations:** 1 Biosciences, College of Health, Medicine and Life Sciences, Brunel University London, Uxbridge UB8 3PH, UK; aeman.zahra@brunel.ac.uk (A.Z.); 1706896@brunel.ac.uk (Q.D.); marcia.hall@nhs.net (M.H.); jeyarooban.jeyaneethi@brunel.ac.uk (J.J.); elisabete.silva@brunel.ac.uk (E.S.); 2 Mount Vernon Cancer Centre, Northwood HA6 2RN, UK

**Keywords:** EDC, BPA, ovarian cancer, biomarker, bioinformatics

## Abstract

Endocrine-disrupting chemicals (EDCs) can exert multiple deleterious effects and have been implicated in carcinogenesis. The xenoestrogen Bisphenol A (BPA) that is found in various consumer products has been involved in the dysregulation of numerous signalling pathways. In this paper, we present the analysis of a set of 94 genes that have been shown to be dysregulated in presence of BPA in ovarian cancer cell lines since we hypothesised that these genes might be of biomarker potential. This study sought to identify biomarkers of disease and biomarkers of disease-associated exposure. In silico analyses took place using gene expression data extracted from The Cancer Genome Atlas (TCGA) and the Genotype-Tissue Expression (GTEx) databases. Differential expression was further validated at protein level using immunohistochemistry on an ovarian cancer tissue microarray. We found that 14 out of 94 genes are solely dysregulated in the presence of BPA, while the remaining 80 genes are already dysregulated (*p*-value < 0.05) in their expression pattern as a consequence of the disease. We also found that seven genes have prognostic power for the overall survival in OC in relation to their expression levels. Out of these seven genes, Keratin 4 (KRT4) appears to be a biomarker of exposure-associated ovarian cancer, whereas Guanylate Binding Protein 5 (GBP5), long intergenic non-protein coding RNA 707 (LINC00707) and Solute Carrier Family 4 Member 11 (SLC4A11) are biomarkers of disease. BPA can exert a plethora of effects that can be tissue- or cancer-specific. Our in silico findings generate a hypothesis around biomarkers of disease and exposure that could potentially inform regulation and policy making.

## 1. Introduction

Endocrine-disrupting chemicals (EDCs) are exogenous substances that can disturb/compromise the normal functions of the endocrine system in both humans and animals and, subsequently, increase the risk of adverse health effects [1]. EDCs are widespread in the environment and can accumulate across the entire food chain, primarily due to their long half-life and the inability of the body to metabolize them [2]. Depending on their origin, EDCs can be subclassified as industrial, agricultural, residential and pharmaceutical [2].

Bisphenol A (BPA) is an EDC that is commonly used as a monomer to manufacture polycarbonate plastics [3]. The world production of BPA is estimated to reach over 7000 thousand tons annually by the end of 2023 [4], making it one of the highest volume chemicals. Its prevalence in numerous commercial products, ranging from food packaging and food contact materials to thermal paper, and medical materials and devices means that humans are exposed to BPA on a daily basis [5]. Previous studies have shown that ingestion of contaminated foods and beverages, as well as inhalation and skin absorption, are common routes of human exposure to this chemical [6]. Environmental factors such as heat or pH can cause leaching of BPA into its surroundings, leading to potential environmental and human exposure, as well as risks to health. Infants aged 0–6 exclusively fed with canned liquid formula and using polycarbonate bottles have been estimated to have highest BPA exposures [7]. As a result, BPA has been found to accumulate in the body with various levels being detected in the adipose tissue [8], serum [9], maternal and fetal plasma [10], breast milk [11], placenta [12] and umbilical cord [9].

At the molecular level, BPA is a xenoestrogen (i.e., it has estrogen-like activity) and therefore can interfere with the estrogen signalling pathways [5,13,14]. The estrogen signalling pathway is regulated at genomic level by estrogen receptors (ERα and ERβ) that can bind to estrogen response elements in the nucleus upon activation and modulate transcriptional responses. In addition, the G protein-coupled receptor 30 (GPR30) mediates the non-genomic signalling of estrogen [15]. GPR30 plays a key role in the physiology of the reproductive system [16,17] and metabolism [18]. In the case of BPA, it has been shown to bind to multiple ERs including ERα, ERβ (cytoplasmic and membrane-bound), GPR30 and human nuclear receptor estrogen-related receptor gamma (ERRγ) [19,20,21,22,23,24,25,26].

There is growing evidence that BPA can affect both male and female reproductive systems resulting in infertility, precocious puberty, endometriosis [27] and even many hormone-dependent malignancies such as breast and prostate cancers [14,28]. Moreover, studies [29,30] have raised the possibility of a direct link between BPA and ovarian cancer, prompting precautionary actions against excess exposure to this EDC [31].

Ovarian cancer (OC) is the sixth most common cancer among females in the UK, accounting for 4% of all new cases of cancer [32]. Every year over 7300 women are diagnosed with ovarian cancer, and it is projected that 10,501 new cases will be diagnosed in the UK in 2035 [32,33]. The rise in cases, as well as the staggering costs of treatment, highlight the need for investigating all the potentially preventable causes for this disease. Earlier studies of the effects of BPA on ovaries have indicated a time-dependent relationship. In particular, the study by Susiarjo et al. [34] on pregnant mice exposed to BPA showed synaptic abnormalities, e.g., partial or complete synaptic failure of a single chromosome pair, end-to-end associations between non-homologous chromosomes and an increased risk of aneuploidy. Treatment of an ERα- and ERβ-positive ovarian cell line with estrogen or BPA altered expression of genes involved in apoptosis, cancer and cell cycle [35]. Further studies have also implicated BPA in ovarian cancer in vitro. Using OVCAR-3, an ovarian cancer cell line, BPA exerted an estrogenic effect stimulating cell migration and up-regulation of certain metalloproteinases and N-cadherin [36]. In the same cell line, BPA increased cell proliferation and decreased activity of the caspase-3 pathway [37].

In this paper, we present the analysis of a set of 94 genes that have been shown to be dysregulated in presence of BPA in OC cell lines [30]. We looked at comparing the expression landscape in ovarian normal tissue and OC under the influence of BPA. We found that 14 out of 94 genes are solely dysregulated in the presence of BPA, while the remaining 80 genes are already dysregulated (*p*-value < 0.05) in their expression pattern, presumably as a consequence of the disease. This study sought to identify biomarkers of disease and associated exposure that could potentially inform regulation and policy making.

## 2. Materials and Methods

### 2.1. Bioinformatics Analysis

#### 2.1.1. Data Availability

The group of 94 genes shown to be dysregulated in the SKOV3 cell line in the presence of BPA was extracted from the published paper by Hui et al., 2018 [30]. SKOV3 cell line is a commonly used cellular model of high-grade serous ovarian cancer (HGSOC). The 94 genes were annotated using information regarding their genomic location, gene name, biotype and Ensembl ID from GeneCards/Ensembl v96.

Gene expression data and sample phenotype information (Table 1) were extracted from the data generated by The Cancer Genome Atlas (TCGA) research network (https://www.cancer.gov/tcga, last accessed on 20 November 2020) and the Genotype-Tissue Expression (GTEx) project (https://www.gtexportal.org, last accessed on 20 November 2020) as published in the Xena repository hosted at the University of California Santa Cruz (UCSC) [38]. Specifically, we analysed data from the TCGA-TARGET-GTEX pan-dataset normalised cohort. The raw RNAseq data from TCGA and GTEx were processed and normalised by the UCSC using the TOIL pipeline, a computation framework that facilitates the quantification of gene expression as well as cross-dataset comparison without any computational batch effects [39]. The gene expression values are presented in units of log_2_(norm_count+ 1). In terms of histological grades, the National Cancer Institute grading system (National Institute of Health, Bethesda, Maryland, USA) was used (i.e., G1–G4) [40].

#### 2.1.2. Functional Analysis

The genes were functionally characterised using Gene Ontology (GO) database [41] as recorded in FunRich (version 3.1.3) software [42]. Seventy-seven (protein-coding genes) of the ninety-four analysed genes were matched in the FunRich, with the remainder 17 having no associated data. The enrichment of the GO terms related to biological processes, biological pathways, molecular functions and expression sites was computed. A threshold *p*-value of 0.05 was used to ascertain the statistical significance of the results.

#### 2.1.3. Immunohistochemistry (IHC)

Immunohistochemistry was used to measure the gene expression at the protein level in tissue samples from ovarian cancer patients (all patient information is given in the Supplementary Table S1). Commercially available ovarian carcinoma tissue arrays, containing 90 cases of ovarian tumour with 10 adjacent normal ovary tissues, single core per case (Biomax, Derwood, MD, USA), were used to examine the expression of SLC4A11 and RARRES3. All tissues were collected under the highest ethical standards with the donor being informed completely and with their consent. Moreover, all human tissues were collected under Health Insurance Portability and Accountability Act (HIPAA) approved protocols. The slides were deparaffinized following a series of washes in Histo-Clear (National Diagnostics) and decreasing concentrations of ethanol. Slides were subsequently boiled in sodium citrate (Merck Life Science UK Ltd, Gillingham, UK) for 20 min using a microwave and cooled down using running tap water for 10 min. The slides were washed twice in phosphate-buffered saline (PBS) with 0.025% *v/v* Triton-X 100 (PBS-T) for 5 min each and further incubated with 3% *v/v* hydrogen peroxide in PBS for 15 min before 3 more washes in PBS-T. The slides were blocked using 5% BSA in PBS for 1 h within a humidity chamber (HC) at room temperature before the addition of primary antibodies to each slide: SLC4A11 (HPA018120—Merck Life Science UK Ltd, Gillingham, UK) and RARRES3 (HPA011219— Merck Life Science UK Ltd, Gillingham, UK) (1:100 dilution in 5% BSA in PBS)—and incubated overnight at 4 °C in the HC. After incubation, the slides were washed 3 times for 5 min each with PBS-T before the addition of anti-rabbit secondary (Zytochem Plus kit), 2BSCIENTIFIC Ltd, Upper Heyford, UK and left to incubate for 1 h at room temperature in the HC. The washes were repeated, and the slides were further incubated with streptavidin–HRP conjugate (Zytochem Plus kit) for 30 min in HC at room temperature. DAB (3,3′-diaminobenzidine) substrate solution (Vector Laboratories, Burlingame, CA, USA) containing hydrogen peroxide was loaded on the slides for 10 min after 3 washes with PBS-T. Slides were washed in H_2_O for 5 min and then incubated with Harris’ haematoxylin for 30 s followed by 0.1% *w/v* sodium bicarbonate for 60 s before dehydration in increasing ethanol concentrations and Histo-Clear. Images of the stained cores were captured using an EOS 1200D camera attached to a light microscope. The images were then analysed under a light microscope giving a score based on how well the cores on the slide were stained (0 = <10% stained, 1 = 10–25% stained, 2 = 25–50% stained, 3 = 50–75% stained and 4 = >75% stained). This was repeated 3 times, and an average was calculated based on the scores for each core.

#### 2.1.4. Statistical Analysis

All data processing and statistical analyses were conducted using R (v. 3.5.2, The R Foundation for Statistical Computing, Vienna, Austria) under R Studio desktop application (version 1.1.463, RStudio, Boston, Massachusetts, USA). Student *t*-test was used to test the statistical significance in the change in expression between two given states (e.g., normal vs. tumour) with a significance threshold set at a *p*-value lower than 0.05. *t*-test was selected as the primary statistics test for normally distributed data. The Kaplan–Meier estimator was used to calculate and analyse the survival of ovarian cancer patients over time in regard to the stage of cancer or expression of genes. Survival analysis was conducted using R library “survminer”. The Pearson correlation coefficient was calculated to estimate the correlation between genes based on their expression pattern in both normal and cancerous ovary tissue.

## 3. Results

### 3.1. Transcriptional and Functional Characterisation

**Figure 1:**
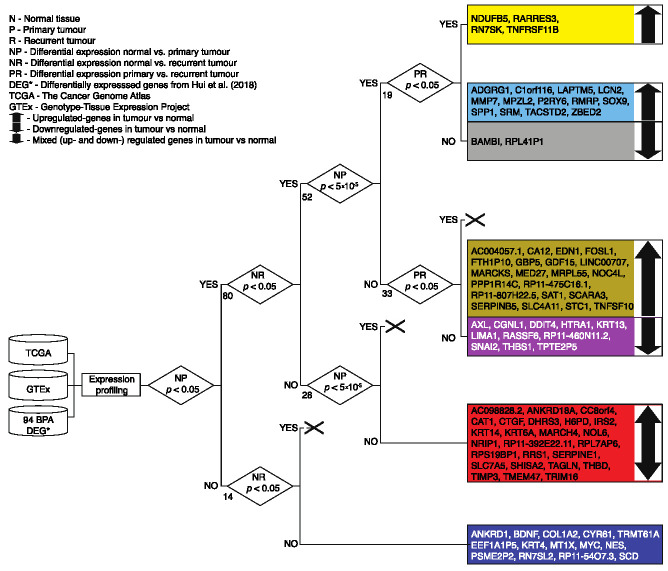
Workflow diagram presenting the data availability, expression analysis and gene selection criteria used in this project. Big black cross represents no gene passing the given condition. The numbers next to the “YES” and “NO” branches indicate the number of genes associated with that condition.

In order to gain a better understanding of the importance and magnitude of the differential expression pattern previously observed for 94 genes in the ovarian cancer cell line SKOV3 under exposure to BPA [30], we set out to analyse the transcriptional landscape of these genes in normal and cancerous ovarian tissues leveraging expression data from unmatched samples from TCGA and GTEx. We computed the *p*-value as a measure of statistical significance for the difference in gene expression levels in three cases: normal vs. primary tumour, normal vs. recurrent tumour and primary vs. recurrent tumour. We selected two thresholds, *p*-value < 0.05 and *p*-value < 0.00005 indicating significant and, respectively, highly significant change in expression, and further differentiated the genes based on whether they were up- or down-regulated. Using these criteria, we were able to distinguish seven gene groups (Figure 1).

Overall, we found 14 genes that show no significant change in expression in tumour samples as compared to controls, hinting that the earlier reported effect of the BPA in ovarian cancer cell line can potentially be regarded as a key driver for some of the associated phenotypical changes (see Figure 1 navy block). At the other end of the spectrum, we identified four genes (yellow block), namely: RNA Component Of 7SK Nuclear Ribonucleoprotein (*RN7SK*), tumour necrosis factor receptor superfamily member 11B (*TNFRSF11B)*, NADH dehydrogenase 1 beta subcomplex 5 (*NDUFB5*) and the retinoic acid receptor responder protein 3 (*RARRES3*). Unsurprisingly, these genes have been previously associated with various malignancies [43,44,45,46,47] including breast and ovarian cancers. The remainder 76 genes were stratified into five groups based on the level of significance in the change of their expression patterns. Thirteen genes (light blue block) were significantly (*p* < 0.05) up-regulated in tumour compared to healthy ovarian tissue. Twenty-two genes (yellow-brown block) were found up-regulated with moderate significant difference (*p* < 0.05) compared to controls. Thirteen genes (grey and purple blocks) were down-regulated in cancer with moderate significant difference. While in the remaining 28 genes in the red block no overall trend was observed, they have statistically high significant difference in primary tumour vs. healthy tissue.

**Figure 2:**
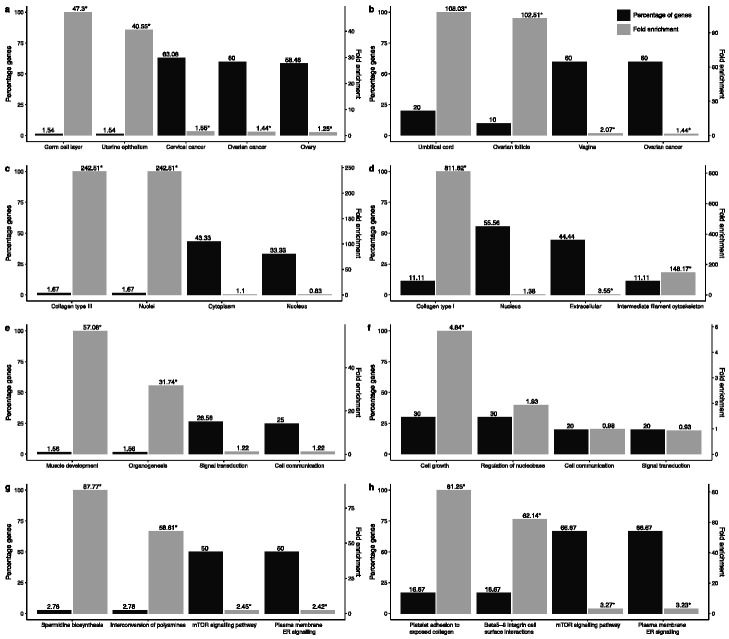
The functional enrichment in Gene Ontology terms in 80 genes (**a**,**c**,**e**,**g**) and 14 genes (**b**,**d**,**f**,**h**) in relation to site of expression (**a**,**b**), cellular components (**c**,**d**), biological processes (**e**,**f**) and biological pathways (**g**,**h**). * *p*-value < 0.05. Mirror figures highlighting the fold enrichment and the gene percentage separately are available in Figures S1 and S2. The genes associated with all the shown phenotypes are given in Table S2.

Next, we looked at functional terms enrichment in the groups of genes that show no change in their transcriptional landscape in cancer (14 genes) as compared to those that do (80 genes). The results are shown in Figure 2.

Gene Ontology analysis results show that majority of genes dysregulated in cancer are enriched in expression sites associated with the female reproductive system. Specifically, the majority of these genes are expressed in ovarian cancer, cervical cancer and normal ovarian tissue, while a small number of genes, namely high-temperature requirement factor A1 (*HTRA1*) and carbonic anhydrase 12 (*CA12*), are highly enriched in the germ cell layer and uterine epithelium. Earlier studies have shown a down-regulation of *HTRA1* in ovarian carcinoma [48] and an up-regulation of the *CA12* gene in breast carcinoma [49]. Cellular components ontology terms enrichment analysis showed that the majority of genes are associated with the cytoplasm and nucleus. Two genes Collagen type III alpha 1 chain (*COL3A1)* and metallothionein 2A (*MT2A*) show a significant fold enrichment in collagen type III and nuclei, respectively. *COL3A1* has been associated with gastric cancer diagnosis, prognosis and therapy [50]. At biological processes level, we see that the majority of genes are involved in signal transduction and cell communication. Significant fold enrichment was observed for transgelin (*TAGLN*) and myelin protein zero-like 2 (*MPZL2*) in relation to organogenesis and muscle development. *MPZL2* has been observed in cell growth, invasion and adhesion of breast cancer cells [51]. Finally, 18 genes, namely *MMP7*, *SPP1*, *SERPINB5*, *FOSL1*, *GDF15*, *EDN1*, *BAMBI*, *DDIT4*, *SNAI2*, *LIMA1*, *KRT14*, *CTGF*, *MT2A*, *NRIP1*, *THBD*, *IRS2*, *SERPINE1* and *TAGLN* are associated with the mTOR signalling and plasma membrane estrogen receptor signalling pathways.

Functional enrichment analysis of the 14 remaining genes revealed that expression sites are enriched for female reproductive systems. Specifically, the majority of these genes (60%) are expressed in the vagina and ovarian cancer, while a small fraction (10–20%) is enriched in terms related to umbilical cord and ovarian follicles. Biological processes terms enrichment analysis showed a third of the genes, namely *COL1A2*, *KRT4*, *NES*, *MYC*, *TRMT61A* and *ANKRD1*, is enriched in cell growth and regulation of nucleobase. From the biological pathway terms enrichment analysis, we observed that the majority of the genes (66.67%), namely *MYC*, *COL1A2*, *CYR61* and *BDNF* are associated with the mTOR pathway and plasma membrane estrogen receptor signalling.

As the GO terms enrichment analysis suggested a couple of major trends, we investigated whether the similarities between genes are preserved at expression level. To this end, we computed the Pearson correlation coefficient for all possible gene pairs using their expression profiles in normal and tumour samples (Figure S3). Overall, we observed a weaker correlation in healthy tissue compared to cancer, suggesting a pervasive expression pattern in tumour mainly driven by the disease state.

**Figure 3:**
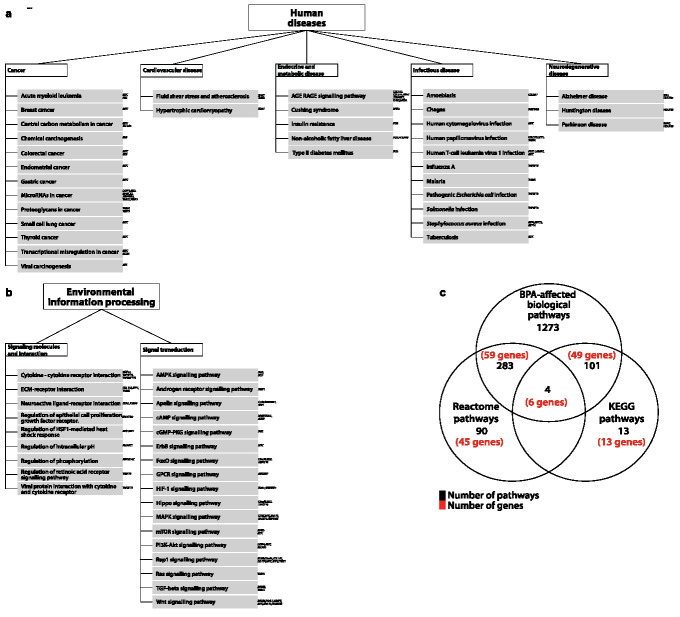
Biological pathways associated with 94 BPA dysregulated genes in humans. (**a**) Human-disease-associated pathways. (**b**) Environmental information processing pathways. (**c**) Venn diagram showing the common pathways in KEGG and Reactome and their intersection with BPA-impacted pathways reported in CTD. Genes that are affecting each pathway are shown on the left corner of each block.

We expanded further the functional analysis by leveraging data on biological pathways from the Kyoto Encyclopedia of Genes and Genome (KEGG), Comparative Toxicogenomics Database (CTD), and Reactome biological data repositories (Figure 3). We found that the 94 genes are mainly involved in pathways associated with human diseases, in particular cancer (Figure 3a) and various infectious diseases (viral, bacterial and parasitic), and environmental information processing (Figure 3b). Furthermore, 388 pathways have been previously described in literature as being impacted by BPA exposure (see Figure 3c).

### 3.2. Evaluation of Prognosis and Diagnosis Potential

**Figure 4:**
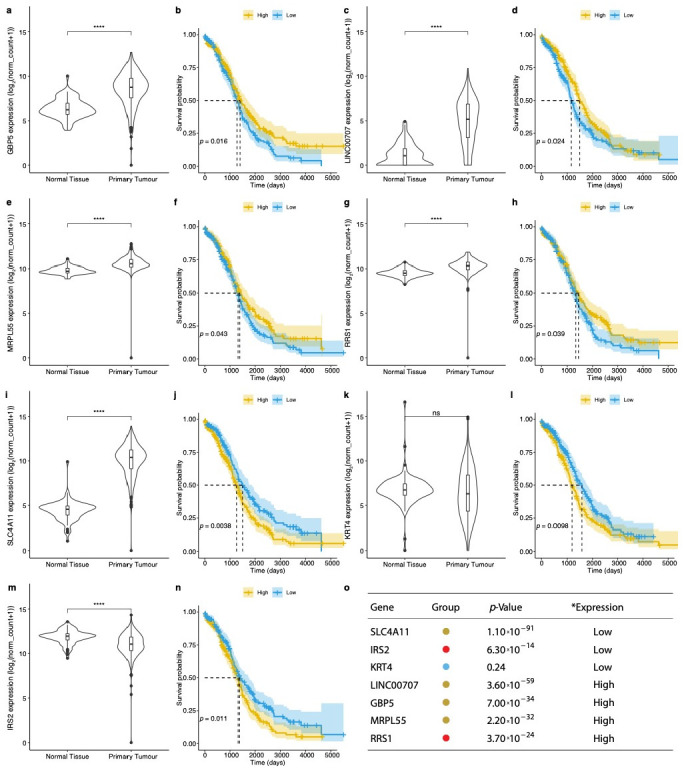
(**a**,**c**,**e**,**g**,**i**,**k**,**m**) Violin plots summarising the distribution of expression values of 7 genes, namely GBP5, LINC00707, MRPL55, RRS1, SLC4A11, KRT4 and IRS2, in normal, primary and recurrent tumour samples. (**b**,**d**,**f**,**h**,**j**,**l**,**n**) KM plots for the overall survival rate for samples stratified based on their expression value. *p*-value indicates the statistically significant difference between patients’ survival stratification by high and low expression groups. **** indicates a significant change in expression with a *p*-value < 5×10^−5^ , ns indicates that there is no significant change in the expression between the two states. Table (**o**) shows genes with a significant change in the OSR with the change in their expression. * Expression associated with higher survival rate. *p*-value indicates the statistically significant difference between OC and normal control. Coloured dots represent a group these genes belong to according to Figure 1.

We evaluated the biomarker potential of the 94 genes by studying the overall survival rate in ovarian cancer patients using the TCGA data in Kaplan-Meyer analysis. We started by examining the baseline survival rate for patients with ovarian cancer by age, stage and disease recurrence observations (Figure S4). As expected, these phenotypes indicated that patients diagnosed at an earlier stage or younger age had a better overall prognosis. However, they provided no indication with respect to the effect of individual gene activity on the survival potential. To this end, we stratified the transcriptional profile of each gene into high and low expression levels using the mean expression value as a discriminant. Overall, we found five up-regulated genes, namely solute carrier family 4 member 11 (*SLC4A11*), guanylate binding protein 5 (*GBP5*), long intergenic non-protein coding RNA 707 (*LINC00707*), mitochondrial ribosomal protein L55 (*MRPL55*) and ribosome biogenesis regulator 1 homolog (*RRS1*), and two down-regulated genes in ovarian cancer, insulin receptor substrate 2 (*IRS2*) and keratin 4 (*KRT4*) that show a statistically significant predictive power for the patient outcome (Figure 4). The seven genes, with the exception of *KRT4*, also show a statistically significant change in expression between the normal and primary tumour samples.

**Figure 5:**
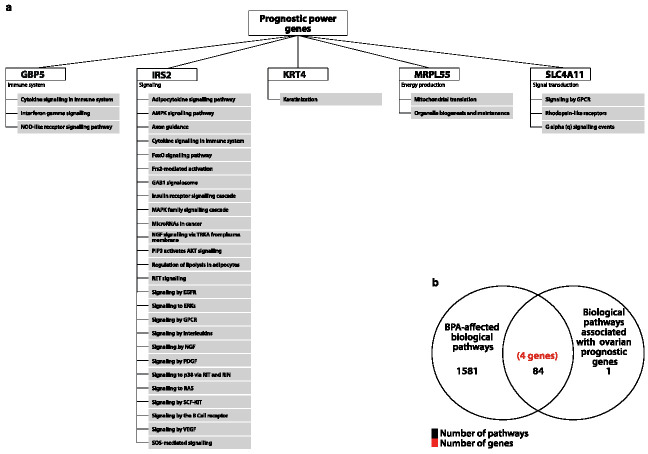
(**a**) represents all possible pathways affected by 5 potential predictive power genes in humans. (**b**) Venn diagram represents all possibly affected pathways upon the exposure of BPA to 7 genes.

In summary, Kaplan-Meyer analysis showed that four genes (*GBP5*, *LINC00707*, *MRPL55*, *RRS1*) are associated with a positive patient outcome when over-expressed, while for the other three (*SLC4A11*, *KRT4* and *IRS2*), their up-regulation is related with a poorer prognosis. It should be noted that the above-mentioned genes are also dysregulated in other cancers, and therefore their prognostic potential might not be limited to ovarian cancer. Similar, the association of high-expression with positive patient outcome has been previously reported for *GBP5* in other cancer types such as skin [52], breast and colorectal cancer [53,54]. Pathway analysis of the five protein-coding genes from this group (Figure 5a) suggests a wide repertoire of roles. For example, GBP5 might play a role in immune responses, MRPL55 in energy production and SLC4A11 in signal transduction mechanisms. The most diverse effects on a variety of signalling pathways implicated in carcinogenesis were exhibited by IRS2. Finally, we looked at the association between the seven prognostic genes and BPA-affected pathways (Figure 5b). We found that earlier studies link four genes (*IRS2*, *KRT4*, *GBP5* and *MRPL55*) with BPA suggesting that exposure to this EDC agent can potentially affect their prognostic power.

**Figure 6:**
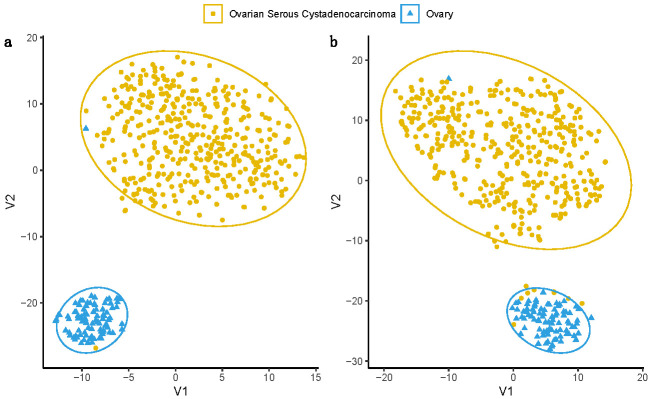
Tumour and normal tissue classification potential revealed by t-distributed stochastic neighbour embedding (t-SNE). Green points represent ovarian tumour samples (*n* = 427), and black points represent ovarian normal tissue samples (*n* = 88). The V1 and V2 are the t-SNE projection axis and do not have a biological meaning. (**a**) represents 94 genes’ expression matrix in TCGA and GTEx embedded using t-SNE. (**b**) represents seven prognostic power gene expression matrix in TCGA and GTEx embedded using t-SNE.

Building on the differential expression analysis, we tested the ovarian cancer diagnostic power for the 94 gene set. To this end, we used t-distributed stochastic neighbour embedding (t-SNE) dimensionality reduction method to discriminate between the normal and tumour samples using the gene expression profiles (Figure 6).

We found that, overall, the 94 genes are an excellent collective ovarian cancer diagnosis biomarker. Given that the data are curated from the ovarian cancer genome sets from GTEx and TGCA, this diagnostic feature might be likely to be for all ovarian cancers, but further research is needed to include a wider repertoire of OC subgroups. Moreover, the seven genes with prognostic power seem to perform also very well in discriminating the healthy and cancerous samples. 

Next, we investigated whether the 94 genes are able to distinguish potential risk groups in the human population. For this, we analysed the t-SNE stratification on a number of factors such as age, race and ethnicity (Figure S5). No statistically significant correlation between the gene expression pattern and the selected phenotypes was observed. Furthermore, the gene transcriptional landscape was also not correlated with the cancer stage. 

**Figure 7:**
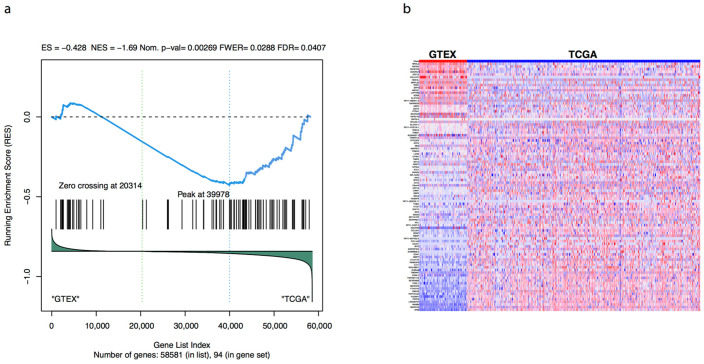
Gene set enrichment analysis for the 94 BPA dysregulated genes. (**a**) Running sum and relative ranks of genes against the human gene set background (58,581 genes). (**b**) Expression dataset sorted by correlation with the phenotype.

We further performed a gene set enrichment analysis to evaluate the relative importance of the genes in the seven groups with respect to the differential expression pattern in tumour (primary and recurrent) compared to normal. We found that the set of 94 genes had a statically significant negative enrichment score, with the bulk of the genes (51) forming the core set of genes that account for the enrichment signal [55] (see Figure 7, Table S3). Furthermore, from the seven genes with biomarker potential, *LINC00707*, *GBP5* and *IRS2* were shown to be key contributors to the enrichment score suggesting a strong association with differential expression in ovarian cancer versus normal.

### 3.3. BPA Effect on Gene Function and Activity

**Figure 8:**
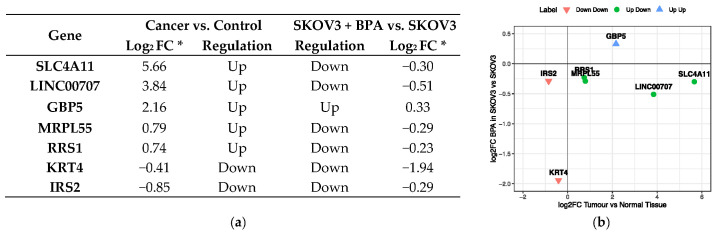
Evaluation of BPA effect on genes with biomarker potential. (**a**) Table of expression changes for tumour tissue and cancer cell line experiments. * FC is the fold change ratio between the two states. (**b**) Scatter plot of the expression changes upon BPA exposure.

The analysis of Hui et al. [30] showed that the environmental dose of BPA can significantly alter the expression of 94 genes in ovarian cancer cell lines. As some of these genes have diagnostic and prognostic power and can be potentially used as clinical biomarkers, it is important to evaluate the effect of low-level (10 nM) BPA exposure of the predictive characteristics. For this, we compared the observed fold change in gene expression between two states in the following two experiments: (1) normal ovarian tissue vs. ovarian cancer (data extracted from TCGA and GTEx) and (2) SKOV3 ovarian cancer cell line in presence and absence of BPA (data extracted from [29]) as shown in Figure 8.

Overall, we found that for three genes, *GBP5*, *LINC00707* and *SLC4A11*, the BPA effect on the expression is substantially smaller compared to the effect observed as a consequence of cancer. Moreover, their collective pattern of expression is a good discriminant between tumour and normal samples (see Figure S6). For *IRS2*, *RRS1* and *MRPL5*, we observed that the fold change in expression is comparable in cancer and under BPA treatment, suggesting that BPA presence can bias the predictive power of these genes. By contrast, we found that BPA exposure is the main driving force for the change in expression in *KRT4*, making it a potential exposure biomarker for BPA. This feature is unique to the keratin 4 among all 94 genes investigated in both its magnitude level and its statistical significance (see Figure S7).

One potential confounding factor is the lack of information regarding the BPA exposure in TCGA and GTEx samples. To address this, we investigated the potential BPA contamination in these datasets by looking at the gene expression rank, where top rank is given to the gene with the highest expression level and the lowest rank to the gene with the lowest expression level (Table S4). We worked under the premise that if a significant number of patients were exposed to BPA under similar levels as those described by Hui et al., when sorting the genes by their expression values, we would observe a similar order to that seen under the BPA influence. We found no significant correlation between the gene expression rank in presence of BPA and the tumour and normal ovarian samples from TCGA and GTEx, respectively. This result suggests that although we cannot establish with confidence whether some samples have been exposed to BPA, overall, the effects can be attributed to the specific genome biology in each case.

### 3.4. Ovarian Cancer Immunohistochemistry Analysis

**Figure 9:**
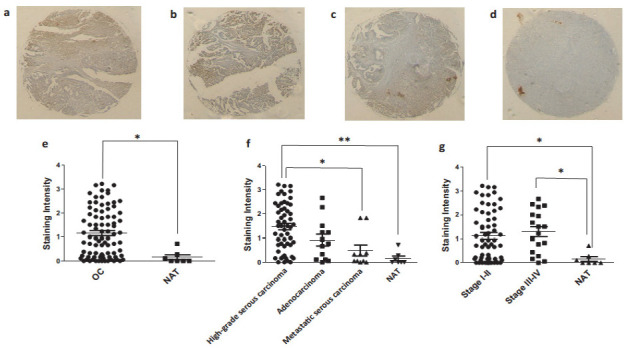
Immunohistochemistry for RARRES3 expression in different pathologies of ovarian tissue array clinical samples: high-grade serous carcinoma (**a**), mucinous adenocarcinoma (**b**), metastatic carcinoma (**c**), normal adjacent tissue (**d**), expression of RARRES3 in ovarian cancer (OC; including high- and low-grade serous carcinoma, mucinous adenocarcinoma, metastatic serous carcinoma) compared to the normal control (**e**), RARRES3 expression in different pathologies of ovarian cancer (**f**) and RARRES3 expression in clinical samples of different stages (**g**). NAT: normal adjacent tissue, * *p*-value < 0.05, ** *p*-value < 0.001.

**Figure 10:**
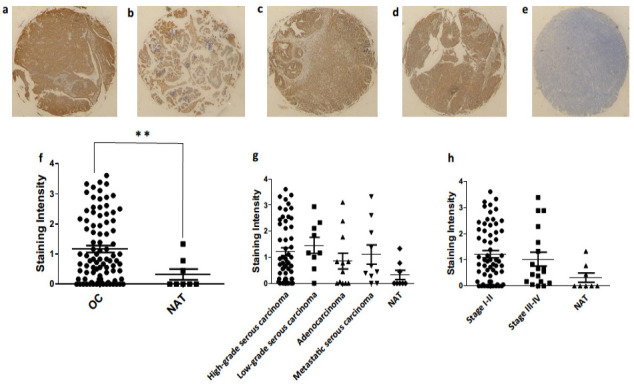
Immunohistochemistry for SLC4A11 expression in different pathologies of ovarian tissue array clinical samples: high-grade serous carcinoma (**a**), low-grade serous carcinoma (**b**), mucinous adenocarcinoma (**c**), metastatic serous carcinoma (**d**), normal adjacent tissue (**e**), expression of SLC4A11 compared to the normal control (**f**), SLC4A11 expression in different pathologies of ovarian cancer (**g**) and RARRES3 expression in clinical samples of different stages (**h**). OC: ovarian cancer (including high- and low-grade serous carcinoma, mucinous adenocarcinoma, metastatic serous carcinoma); NAT: normal adjacent tissue, ** *p*-value < 0.001.

In order to validate our in silico data and identify any changes in protein expression with respect to type or stage of the disease, we used an ovarian cancer tissue array to perform immunohistochemistry in a number of clinical samples (90 ovarian cancer patients’ data and 10 normal adjacent controls). We validated the expression of *RARRES3* (in Figure 9) and *SLC4A11* (in Figure 10). These genes were selected as representatives of the highly significant up-regulated genes in the ovarian cancer and the biomarker groups, respectively.

*RARRES3* was expressed in high-grade serous carcinoma, mucinous adenocarcinoma and metastatic serous carcinoma (Figure 9a–c). Statistical analysis on *RARRES3* revealed that despite the interpatient variation, OC patients expressed more *RARRES3* (*p*-value < 0.05) at protein level when compared to normal adjacent control tissue (NAT) as shown in (Figure 9e). We observed from Figure 9f that change in the expression of *RARRES3* is significantly up-regulated (** *p*-value < 0.001) in high-grade serous carcinoma compared to NAT and metastatic serous carcinoma (* *p*-value < 0.05). When OC patients were grouped in early stages (I and II) and late (III and IV), no apparent differences in the expression of RARRES3 protein were evident. However, RARRES3 was over-expressed in both groups compared to NAT (* *p*-value < 0.05) as shown in Figure 9g.

*SLC4A11* was expressed in high-grade serous carcinoma, low-grade serous carcinoma, mucinous adenocarcinoma and metastatic serous carcinoma (as shown in Figure 10a–d). Here we may infer that high *SLC4A11* expression can be a potential predictor for poor overall survival in low-grade serous ovarian carcinoma. Scoring of immunostaining revealed an apparent difference in the *SLC4A11* expression compared to the normal control (Figure 10f–g), thus corroborating the gene expression reported through data analysis. We then measured *SLC4A11* expression in clinical samples of different stages: I, II, III and IV (Figure 10h). It is also evident that despite the interpatient variation, expression of *SLC4A11* is highly significant (** *p*-value = 0.0074) in OC patients at protein level when compared to NAT (see Figure 10f). However, no significant change was observed between different types and stages of ovarian cancer.

## 4. Discussion

Here we provide a detailed analysis of the functional and activity landscape in ovarian cancer for a set of 94 genes that have been previously shown to be dysregulated under exposure to environmental levels of BPA in ovarian cancer cell lines. Apart from genetic influences on the development of malignancies, other environmental factors such as EDCs may also be an important determinant [56]. However, to date, availability of biomarkers of exposure specific to ovarian cancer is very limited.

We showed that 14 genes do not exhibit any significant changes in tumour compared to normal tissue, and thus the effects observed under BPA treatment can be regarded as the key driving forces for the associated phenotypes. The majority of the genes, however, showed a statistically significant differential expression pattern in cancer, hinting that a combined BPA tumour effect can play a key role in the future development of the disease. Specifically, four genes (*RN7SK*, *TNFRSF11B*, *NDUFB5* and *RARRES3*) were shown to be progressively up-regulated in primary and recurrent tumours compared to normal. These results are in accord with previous reports indicating these genes are highly dysregulated in a variety of diseases [43,44,45]. For example, *TNFRSF11B* exhibited a cancer-specific behaviour in ovarian cancer by contrast to breast, where it was found to be down-regulated and was proposed as a potential prognostic biomarker [57]. Our data suggest that while *TNFRSF11B* can potentially exhibit diagnostic potential, even differentiating between primary and recurrent tumours, it does not have any predictive power for the overall patient outcome.

Gene Ontology analysis of the 80 genes revealed interesting targets in relation to site of expression (e.g., ovarian cancer, cervical cancer and normal ovarian tissue), cellular components (primarily cytoplasm and nucleus), biological processes (e.g., signal transduction) and biological pathways (mainly mTOR and plasma membrane estrogen receptor signalling pathways). Both of these signalling pathways have been implicated in ovarian cancer. The mTOR pathway is a central regulator of cellular events such as proliferation, apoptosis and angiogenesis gauging external energy, growth factor and stress signals with the PI3K/AKT/mTOR pathway being a highly activated cellular signalling pathway in advanced ovarian cancer [58,59,60]. Similarly, there is evidence of involvement of the membrane-bound estrogen receptor GPR30 in cancer [61]. As mentioned, GPR30 can drive genomic and non-genomic events upon activation with estrogen or other estrogen-like compounds such as BPA [62,63].

On the other hand, functional enrichment analysis of the 14 genes revealed that expression sites are enriched for ovarian cancer, vagina and umbilical cord. Similarly, to the 80 genes in question, the genes including *MYC*, *COL1A2*, *CYR61* and *BDNF* are associated with the mTOR pathway and plasma membrane estrogen receptor signalling. Of note, extensive copy number alterations of MYC proto-oncogene BHLH transcription factor *(MYC)* have been observed in high-grade serous ovarian cancer [64], whereas *BDNF* appears to play a role in ovarian cancer, cell migration and angiogenesis [65] and cysteine-rich angiogenic inducer 61 (*CYR61*) is a potential biomarker for prognostic insinuations of ovarian carcinoma [66]. Kaplan–Meyer analysis enabled us to identify seven genes (*GBP5*, *LINC00707*, *MRPL55*, *RRS1*, *SLC4A11*, *KRT4* and *IRS2)* with overall prognostic biomarker potential. The majority of genes displayed a varied phenotype schema: up-regulated in cancer, with positive outcome on up-regulation; up-regulated in cancer, with negative outcome on up-regulation; and down-regulated in cancer, with negative outcome on up-regulation. Next, using the t-SNE dimensionality reduction analysis method, we showed that the combined predictive power of the seven genes results in a strong collective diagnostic marker, suggesting that the seven genes can be used clinically as a cancer panel for both diagnosis and prognosis. However, the selected seven genes could not provide any information regarding population at risk.

Given the fact that all these genes were previously highlighted as having a differential expression pattern under BPA treatment, we investigated further which genes can be suitable candidates for biomarkers of exposure and biomarkers of disease. By evaluating the fold change in expression between normal and primary tumours and comparing it to the fold change between expression in SKOV3 cell line in presence and absence of low-dose BPA, we were able to further stratify the seven genes into three groups. We found that for *GBP5*, *LINC00707* and *SLC4A11*, the effect of BPA exposure is minimal with a potential positive bias in *GBP5* and negative bias in *LINC00707* and *SLC4A11.* By contrast, *KRT4* was shown to be strongly and negatively impacted by BPA exposure, suggesting that BPA can alter the predictive outcome of *KRT4*. Of note, KRT4 shows a particular behaviour exhibiting no significant change in expression between normal and primary tumours but showing a strong positive patient outlook upon down-regulation. Finally, for *IRS2*, *RRS2* and *MRPL5*, we found comparable effects on gene expression under tumour conditions or exposure to BPA. Collectively, these results suggest that a conservative functional cancer panel formed by *GBP5*, *LINC00707* and *SLC4A11* can provide useful insights regarding the diagnosis and overall survival prognosis regardless of the status of BPA exposure of the patient (i.e., biomarkers of disease), while *KRT4* can act as a marker for exposure-associated disease.

The finding that KRT4 can be a potential biomarker of BPA exposure-associated ovarian cancer is of increasing importance given that this gene appears to be under the influence of estrogenic responses. Indeed, estrogens play an important role in the development and growth of ovarian cancer as well as in its subsequent metastatic events. When ER-positive ovarian cancer cells were treated with E2, KRT4 expression was dramatically down-regulated [67,68]. Moreover, when estrogen receptor β (ERβ) was silenced in breast cancer MDA-MB-231 cells, KRT4 expression was significantly increased [69]. When p53 null mammary epithelial cells were treated with the selective estrogen receptor modulator Tamoxifen, it led to a significant up-regulation of KRT4 [70]. Nguyen et al. suggested a functional interplay between Zinc-finger protein 217 (ZNF217) and ERα exists in breast cancer [71]. Interestingly, when ZNF217 is silenced in ovarian cancer in vitro, the KRT4 gene was also significantly down-regulated [72]. A direct link between BPA and KRT4 comes from an in vivo study, where KRT4 promoter was hypomethylated in two-week mice following BPA treatment in utero [73].

In summary, leveraging the available RNAseq data from TCGA and GTEx, we were able to identify a number of new potential biomarkers of exposure-associated disease and biomarkers of diagnostic/prognostic potential for ovarian cancer. Future studies should concentrate on elucidating the impact of BPA on normal ovarian function and correlating the biomarker potential of the above-mentioned genes with clinical data. It would be of interest to measure circulating BPA levels in patients and correlate these concentrations with expression of certain genes, especially KRT4 in both tissue and liquid biopsies. Ultimately, these data can be used to put in place preventative measures to reduce exposure to BPA that consequently might impact disease progression.

## Figures and Tables

**Table 1:** Data summary for the normal ovarian tissue and ovarian cancer samples from TCGA and GTEx datasets. NOS: not otherwise specified; NA: not applicable; FNA: fine-needle aspiration; GB: grade borderline; GX: grade cannot be assessed.

Phenotype	TCGA	GTEx
**Total Samples**	427	88
Normal tissue	-	88 (100%)
Primary tumour	419 (98.13%)	-
Recurrent tumour	8 (1.87%)	-
**Category**		
Normal ovary	-	
Ovarian serous	427 (100%)	88 (100%)
Cystadenocarcinoma		NA
**Primary diagnosis**		NA
Serous cystadenocarcinoma, NOS	422 (98.83%)	
Papillary serous cystadenocarcinoma	4 (0.94%)	
Cystadenocarcinoma, NOS	1 (0.23%)	
**Clinical stage**		NA
Stage I	1 (0.23%)	
Stage II	26 (6.09%)	
Stage III	334 (78.22%)	
Stage IV	63 (14.75%)	
**Overall survival (days)**	Min 8 Max 5481	NA
**Age range (years)**	30–87	20–69
Age < 50	103 (24.12%)	39 (44.31%)
Age > 50	324 (75.88%)	49(55.68%)
**Mortality**		NA
Living	162 (37.94%)	
Deceased	265 (62.06%)	
**Initial Diagnosis Methods**		NA
Cytology (e.g., pleural fluid)	54 (12.65%)	
Excisional biopsy	5 (1.17%)	
FNA biopsy	9 (2.11%)	
Incisional biopsy	6 (1.41%)	
Tumour resection	347 (81.26%)	
Unspecified method	6 (1.41%)	
**Neoplasm Histologic Grade**		NA
G1	1 (0.23%)	
G2	52 (12.18%)	
G3	363 (85.01%)	
G4	1 (0.23%)	
GB	2 (0.47%)	
GX	6 (1.41%)	
Unspecified grade	2 (0.47%)	

## Data Availability

All data used in this paper is publicly available through the TCGA, GTEx and GEO databases.

## Supplementary Materials

The following are available online at https://www.mdpi.com/article/10.3390/jcm10091979/s1, Figure S1. The functional enrichment in gene ontology terms in 14 genes in relation to site of expression (a,b), cellular components (c,d), biological processes (e,f) and biological pathways (g,h). **p*-val < 0.05. Figure S2. The functional enrichment in gene ontology terms in 80 genes in relation to site of expression (a,b), cellular components (c,d), biological processes (e,f) and biological pathways (g,h). **p*-val < 0.05. Figure S3. Heatmap of 94 genes in (a) normal ovarian tissue and (b) tumorous ovarian tissue showing correlation between these genes. Deep dark blue colour shows a strong correlation, while deep red colour shows no correlation. Figure S4. KM-plots for stratifying by (a) stage (late – III& IV vs early – I&II), (b) age (late – >60 vs early – <60), and (c) recurrent disease (yes vs no). Figure S5. tSNE discrimination between various phenotypes using the information from the 94 gene expression profiles. Figure S6. tSNE discrimination between tumour and normal samples using the information from the GBP5, SCL4A11 and LINC0070 gene expression profiles. Figure S7. Scatter plot of the expression changes upon BPA exposure as compared to the changes in expression driven by ovarian cancer alone. The labels indicate the pairing in the change in expression in cancer as in SKOV3 cell lines under BPA treatment as compared to their respective controls. The colours are indicative of the statistical significance of the change in expression in ovarian tumor samples vs normal healthy tissue. Table S1. Details of the clinicopathological features of the tissues used for the microarray. Table S2. List of genes associated with the phenotypes in Figure 2. Table S3. Gene set enrichment analysis results for 94 BPA dysregulated genes. Table S4. Gene expression rank in Hui et al, TCGA, and GTEx datasets.

## References

[1] La Merrill M.A., Vandenberg L.N., Smith M.T., Goodson W., Browne P., Patisaul H.B., Guyton K.Z., Kortenkamp A., Cogliano V.J., Woodruff T.J. (2020). Consensus on the key characteristics of endocrine-disrupting chemicals as a basis for hazard identification. Nat. Rev. Endocrinol..

[2] Lauretta R., Sansone A., Sansone M., Romanelli F., Appetecchia M. (2019). Endocrine Disrupting Chemicals: Effects on Endocrine Glands. Front. Endocrinol..

[3] Wang Z., Liu H., Liu S. (2016). Low-Dose Bisphenol A Exposure: A Seemingly Instigating Carcinogenic Effect on Breast Cancer. Adv. Sci..

[4] Global Bisphenol A Market Report 2018: Analysis 2013–2017 & Forecasts 2018–2023. https://www.prnewswire.com/news-releases/global-bisphenol-a-market-report-2018-analysis-2013-2017--forecasts-2018-2023-300757673.html.

[5] Alavian-Ghavanini A., Lin P.-I., Lind P.M., Rimfors S.R., Lejonklou M.H., Dunder L., Tang M., Lindh C., Bornehag C.-G., Rüegg J. (2018). Prenatal Bisphenol A Exposure is Linked to Epigenetic Changes in Glutamate Receptor Subunit Gene Grin2b in Female Rats and Humans. Sci. Rep..

[6] Vandenberg L.N., Colborn T., Hayes T.B., Heindel J.J., Jacobs D.R., Lee D.-H., Myers J.P., Shioda T., Soto A.M., Saal F.S.V. (2013). Regulatory decisions on endocrine disrupting chemicals should be based on the principles of endocrinology. Reprod. Toxicol..

[7] Ottawa C. Toxicological and Health Aspects of Bisphenol A Report of Joint FAO/WHO Expert Meeting and Report of Stakeholder Meeting on Bisphenol A Food and Agriculture Organization of the United Nations. www.who.int.

[8] Artacho-Cordón F., Fernández M., Frederiksen H., Iribarne-Durán L., Jiménez-Díaz I., Vela-Soria F., Andersson A., Martin-Olmedo P., Peinado F., Olea N. (2018). Environmental phenols and parabens in adipose tissue from hospitalized adults in Southern Spain. Environ. Int..

[9] Lee J., Choi K., Park J., Moon H.-B., Choi G., Lee J.J., Suh E., Kim H.-J., Eun S.-H., Kim G.-H. (2018). Bisphenol A distribution in serum, urine, placenta, breast milk, and umbilical cord serum in a birth panel of mother–neonate pairs. Sci. Total Environ..

[10] Zbucka-Krętowska M., Łazarek U., Miltyk W., Sidorkiewicz I., Pierzyński P., Milewski R., Wołczyński S., Czerniecki J. (2019). Simultaneous analysis of bisphenol A fractions in maternal and fetal compartments in early second trimester of pregnancy. J. Périnat. Med..

[11] Tateoka Y. (2014). Bisphenol A Concentration in Breast Milk following Consumption of a Canned Coffee Drink. J. Hum. Lact..

[12] Strakovsky R.S., Schantz S.L. (2018). Impacts of bisphenol A (BPA) and phthalate exposures on epigenetic outcomes in the human placenta. Environ. Epigenetics.

[13] Vandenberg L.N., Maffini M.V., Sonnenschein C., Rubin B.S., Soto A.M. (2009). Bisphenol-A and the Great Divide: A Review of Controversies in the Field of Endocrine Disruption. Endocr. Rev..

[14] Rochester J.R. (2013). Bisphenol A and human health: A review of the literature. Reprod. Toxicol..

[15] Wei W., Chen Z.-J., Zhang K.-S., Yang X.-L., Wu Y.-M., Chen X.-H., Huang H.-B., Liu H.-L., Cai S.-H., Du J. (2014). The activation of G protein-coupled receptor 30 (GPR30) inhibits proliferation of estrogen receptor-negative breast cancer cells in vitro and in vivo. Cell Death Dis..

[16] Kim M.-J., Kim T.-H., Lee H.-H. (2015). G-protein Coupled Estrogen Receptor (GPER/GPR30) and Women’s Health. J. Menopausal Med..

[17] Qian H., Xuan J., Liu Y., Shi G. (2016). Function of G-Protein-Coupled Estrogen Receptor-1 in Reproductive System Tumors. J. Immunol. Res..

[18] Sharma G., Prossnitz E.R. (2016). GPER/GPR30 Knockout Mice: Effects of GPER on Metabolism. Methods in Molecular Biology.

[19] Alonso-Magdalena P., Laribi O., Ropero A.B., Fuentes E., Ripoll C., Soria B., Nadal A. (2005). Low Doses of Bisphenol A and Diethylstilbestrol Impair Ca^2+^ Signals in Pancreatic α-Cells through a Nonclassical Membrane Estrogen Receptor within Intact Islets of Langerhans. Environ. Health Perspect..

[20] Nadal A., Ropero A.B., Laribi O., Maillet M., Fuentes E., Soria B. (2000). Nongenomic actions of estrogens and xenoestrogens by binding at a plasma membrane receptor unrelated to estrogen receptor alpha and estrogen receptor beta. Proc. Natl. Acad. Sci. USA.

[21] Delfosse V., Grimaldi M., Pons J.-L., Boulahtouf A., Le Maire A., Cavailles V., Labesse G., Bourguet W., Balaguer P. (2012). Structural and mechanistic insights into bisphenols action provide guidelines for risk assessment and discovery of bisphenol A substitutes. Proc. Natl. Acad. Sci. USA.

[22] Ali S., Steinmetz G., Montillet G., Perrard M.-H., Loundou A., Durand P., Guichaoua M.-R., Prat O. (2014). Exposure to Low-Dose Bisphenol A Impairs Meiosis in the Rat Seminiferous Tubule Culture Model: A Physiotoxicogenomic Approach. PLoS ONE.

[23] Buoso E., Masi M., Galbiati V., Maddalon A., Iulini M., Kenda M., Dolenc M.S., Marinovich M., Racchi M., Corsini E. (2020). Effect of estrogen-active compounds on the expression of RACK1 and immunological implications. Arch. Toxicol..

[24] Buoso E., Masi M., Racchi M., Corsini E. (2020). Endocrine-Disrupting Chemicals’ (EDCs) Effects on Tumour Microenvironment and Cancer Progression: Emerging Contribution of RACK1. Int. J. Mol. Sci..

[25] Huang W., Ai W., Lin W., Fang F., Wang X., Huang H., Dahlgren R.A., Wang H. (2020). Identification of receptors for eight endocrine disrupting chemicals and their underlying mechanisms using zebrafish as a model organism. Ecotoxicol. Environ. Saf..

[26] Kahn L.G., Philippat C., Nakayama S.F., Slama R., Trasande L. (2020). Endocrine-disrupting chemicals: Implications for human health. Lancet Diabetes Endocrinol..

[27] Wen X., Xiong Y., Jin L., Zhang M., Huang L., Mao Y., Zhou C., Qiao Y., Zhang Y. (2020). Bisphenol A Exposure Enhances Endometrial Stromal Cell Invasion and Has a Positive Association with Peritoneal Endometriosis. Reprod. Sci..

[28] Matuszczak E., Komarowska M.D., Debek W., Hermanowicz A. (2019). The Impact of Bisphenol A on Fertility, Reproductive System, and Development: A Review of the Literature. Int. J. Endocrinol..

[29] Hoffmann M., Rak A., Ptak A. (2018). Bisphenol A and its derivatives decrease expression of chemerin, which reverses its stimulatory action in ovarian cancer cells. Toxicol. Lett..

[30] Hui L., Li H., Lu G., Chen Z., Sun W., Shi Y., Fu Z., Huang B., Zhu X., Lu W. (2018). Low Dose of Bisphenol A Modulates Ovarian Cancer Gene Expression Profile and Promotes Epithelial to Mesenchymal Transition via Canonical Wnt Pathway. Toxicol. Sci..

[31] Caserta D., Di Segni N., Mallozzi M., Giovanale V., Mantovani A., Marci R., Moscarini M. (2014). Bisphenol a and the female reproductive tract: An overview of recent laboratory evidence and epidemiological studies. Reprod. Biol. Endocrinol..

[32] Ovarian Cancer Incidence Statistics Cancer Research UK. https://www.cancerresearchuk.org/health-professional/cancer-statistics/statistics-by-cancer-type/ovarian-cancer/incidence#heading-Zero.

[33] Menon U., McGuire A.J., Raikou M., Ryan A., Davies S.K., Burnell M., Gentry-Maharaj A., Kalsi J.K., Singh N., Amso N.N. (2017). The cost-effectiveness of screening for ovarian cancer: Results from the UK Collaborative Trial of Ovarian Cancer Screening (UKCTOCS). Br. J. Cancer.

[34] Susiarjo M., Hassold T.J., Freeman E., Hunt P.A. (2007). Bisphenol A Exposure in Utero Disrupts Early Oogenesis in the Mouse. PLoS Genet..

[35] Hwang K.-A., Park S.-H., Yi B.-R., Choi K.-C. (2011). Gene Alterations of Ovarian Cancer Cells Expressing Estrogen Receptors by Estrogen and Bisphenol A Using Microarray Analysis. Lab. Anim. Res..

[36] Ptak A., Hoffmann M., Gruca I., Barć J. (2014). Bisphenol A induce ovarian cancer cell migration via the MAPK and PI3K/Akt signalling pathways. Toxicol. Lett..

[37] Ptak A., Wróbel A., Gregoraszczuk E.L. (2011). Effect of bisphenol-A on the expression of selected genes involved in cell cycle and apoptosis in the OVCAR-3 cell line. Toxicol. Lett..

[38] Goldman M., Craft B., Brooks A., Zhu J., Haussler D. (2018). The UCSC Xena platform for public and private cancer genomics data visualization and interpretation. bioRxiv.

[39] Vivian J., Rao A.A., Nothaft F.A., Ketchum C., Armstrong J., Novak A., Pfeil J., Narkizian J., DeRan A.D., Musselman-Brown A. (2017). Toil enables reproducible, open source, big biomedical data analyses. Nat. Biotechnol..

[40] Edge S.B., Compton C.C. (2010). The American Joint Committee on Cancer: The 7th Edition of the AJCC Cancer Staging Manual and the Future of TNM. Ann. Surg. Oncol..

[41] Ashburner M. (2000). Gene ontology: Tool for the unification of biology. Nat. Genet..

[42] Pathan M., Keerthikumar S., Ang C.-S., Gangoda L., Quek C.Y.J., Williamson N.A., Mouradov D., Sieber O.M., Simpson R.J., Salim A. (2015). FunRich: An open access standalone functional enrichment and interaction network analysis tool. Proteomics.

[43] Xie Y., Zheng L., Tao L. (2019). Downregulation of IQGAP2 Correlates with Prostate Cancer Recurrence and Metastasis. Transl. Oncol..

[44] Luan F., Li X., Cheng X., Huangfu L., Han J., Guo T., Du H., Wen X., Ji J. (2020). TNFRSF11B activates Wnt/β-catenin signaling and promotes gastric cancer progression. Int. J. Biol. Sci..

[45] Morales M., Arenas E.J., Urosevic J., Guiu M., Fernández E., Planet E., Fenwick R.B., Fernández-Ruiz S., Salvatella X., Reverter D. (2014). RARRES 3 suppresses breast cancer lung metastasis by regulating adhesion and differentiation. EMBO Mol. Med..

[46] Song H., Sun W., Ye G., Ding X., Liu Z., Zhang S., Xia T., Xiao B., Xi Y., Guo J. (2013). Long non-coding RNA expression profile in human gastric cancer and its clinical significances. J. Transl. Med..

[47] Lu X., Lu J., Liao B., Li X., Qian X., Li K. (2017). Driver pattern identification over the gene co-expression of drug response in ovarian cancer by integrating high throughput genomics data. Sci. Rep..

[48] Gagné A., Têtu B., Orain M., Turcotte S., Plante M., Grégoire J., Renaud M.-C., Bairati I., Trudel D. (2018). HtrA1 expression and the prognosis of high-grade serous ovarian carcinoma: A cohort study using digital analysis. Diagn. Pathol..

[49] Davidson B., Stavnes H.T., Holth A., Chen X., Yang Y., Shih I.M., Wang T.L. (2011). Gene expression signatures differentiate ovarian/peritoneal serous carcinoma from breast carcinoma in effusions. J. Cell. Mol. Med..

[50] Hao S., Lv J., Yang Q., Wang A., Li Z., Guo Y., Zhang G. (2019). Identification of Key Genes and Circular RNAs in Human Gastric Cancer. Med. Sci. Monit..

[51] Wang F., Wang L., Pan J. (2014). PACE4 regulates proliferation, migration and invasion in human breast cancer MDA-MB-231 cells. Mol. Med. Rep..

[52] Wang Q., Wang X., Liang Q., Wang S., Xiwen L., Pan F., Chen H., Li D. (2018). Distinct prognostic value of mRNA expression of guanylate-binding protein genes in skin cutaneous melanoma. Oncol. Lett..

[53] Godoy P., Cadenas C., Hellwig B., Marchan R., Stewart J., Reif R., Lohr M., Gehrmann M., Rahnenführer J., Schmidt M. (2012). Interferon-inducible guanylate binding protein (GBP2) is associated with better prognosis in breast cancer and indicates an efficient T cell response. Breast Cancer.

[54] Tretina K., Park E.-S., Maminska A., MacMicking J.D. (2019). Interferon-induced guanylate-binding proteins: Guardians of host defense in health and disease. J. Exp. Med..

[55] Fleming D.S., Miller L.C. (2016). Leading edge analysis of transcriptomic changes during pseudorabies virus infection. Genom. Data.

[56] Steckling N., Gotti A., Bose-O’Reilly S., Chapizanis D., Costopoulou D., De Vocht F., Garí M., Grimalt J.O., Heath E., Hiscock R. (2018). Biomarkers of exposure in environment-wide association studies—Opportunities to decode the exposome using human biomonitoring data. Environ. Res..

[57] Lu G., Fan L., Zhong X., Yang H., Xie R., Lv Z., Fu D., Luo P., Ma Y. (2017). Dysregulation of TMPRSS3 and TNFRSF11B correlates with tumorigenesis and poor prognosis in patients with breast cancer. Oncol. Rep..

[58] Rogers-Broadway K., Kumar J., Sisu C., Wander G., Mazey E., Jeyaneethi J., Pados G., Tsolakidis D., Klonos E., Grunt T. (2018). Differential expression of mTOR components in endometriosis and ovarian cancer: Effects of rapalogues and dual kinase inhibitors on mTORC1 and mTORC2 stoichiometry. Int. J. Mol. Med..

[59] Rogers-Broadway K.-R., Chudasama D., Pados G., Tsolakidis D., Goumenou A., Hall M., Karteris E. (2016). Differential effects of rapalogues, dual kinase inhibitors on human ovarian carcinoma cells in vitro. Int. J. Oncol..

[60] Xiao Y., Yu Y., Jiang P., Li Y., Wang C., Zhang R. (2020). The PI3K/mTOR dual inhibitor GSK458 potently impedes ovarian cancer tumorigenesis and metastasis. Cell. Oncol..

[61] Langdon S.P., Herrington C.S., Hollis R.L., Gourley C. (2020). Estrogen Signaling and Its Potential as a Target for Therapy in Ovarian Cancer. Cancers.

[62] Prossnitz E.R., Arterburn J.B., Smith H.O., Oprea T.I., Sklar L.A., Hathaway H.J. (2008). Estrogen Signaling through the Transmembrane G Protein–Coupled Receptor GPR30. Annu. Rev. Physiol..

[63] Hafezi S.A. (2019). The Endocrine Disruptor Bisphenol A (BPA) Exerts a Wide Range of Effects in Carcinogenesis and Response to Therapy. Curr. Mol. Pharmacol..

[64] Zeng M., Kwiatkowski N.P., Zhang T., Nabet B., Xu M., Liang Y., Quan C., Wang J., Hao M., Palakurthi S. (2018). Targeting MYC dependency in ovarian cancer through inhibition of CDK7 and CDK12/13. eLife.

[65] Au C.W., Siu M.K., Liao X., Wong E.S., Ngan H.Y., Tam K.F., Chan D.C., Chan Q.K., Cheung A.N. (2009). Tyrosine kinase B receptor and BDNF expression in ovarian cancers—Effect on cell migration, angiogenesis and clinical outcome. Cancer Lett..

[66] Shen H., Cai M., Zhao S., Wang H., Li M., Yao S., Jiang N. (2014). CYR61 overexpression associated with the development and poor prognosis of ovarian carcinoma. Med. Oncol..

[67] O’Donnell A.J.M., Macleod K.G., Burns D.J., Smyth J.F., Langdon S.P. (2005). Estrogen receptor-α mediates gene expression changes and growth response in ovarian cancer cells exposed to estrogen. Endocr. Relat. Cancer.

[68] Walker G., MacLeod K., Williams A.R., Cameron D.A., Smyth J.F., Langdon S.P. (2007). Estrogen-regulated gene expression predicts response to endocrine therapy in patients with ovarian cancer. Gynecol. Oncol..

[69] Schüler-Toprak S., Häring J., Inwald E.C., Moehle C., Ortmann O., Treeck O. (2016). Agonists and knockdown of estrogen receptor β differentially affect invasion of triple-negative breast cancer cells in vitro. BMC Cancer.

[70] Palaniappan M., Edwards D., Creighton C.J., Medina D., Conneely O.M. (2018). Reprogramming of the estrogen responsive transcriptome contributes to tamoxifen-dependent protection against tumorigenesis in the p53 null mammary epithelial cells. PLoS ONE.

[71] Nguyen N.T., Vendrell J.A., Poulard C., Győrffy B., Goddard-Leon S., Bieche I., Corbo L., Le Romancer M., Bachelot T., Treilleux I. (2014). A functional interplay between ZNF217 and Estrogen Receptor alpha exists in luminal breast cancers. Mol. Oncol..

[72] Zhong M., Sun G., Qin J., Qiu Y., Gao Y., Yu Y., Deng Q. (2009). Microarray analysis of gene expression in the ovarian cancer cell line HO-8910 with silencing of the ZNF217 gene. Mol. Med. Rep..

[73] Jorgensen E.M., Alderman M.H., Taylor H.S. (2016). Preferential epigenetic programming of estrogen response after in utero xenoestrogen (bisphenol-A) exposure. FASEB J..

