# Supplementary material for: Identification of Potential Bisphenol A (BPA) Exposure Biomarkers in Ovarian Cancer
Source: J Clin Med. 2021 May 5;10(9):1979. doi: 10.3390/jcm10091979 (PMC8125610; doi:10.3390/jcm10091979)
Supplement: Supplementary file 1 [file jcm-10-01979-s001.zip › jcm-1181876-supplementary.pdf]

Supplementary information

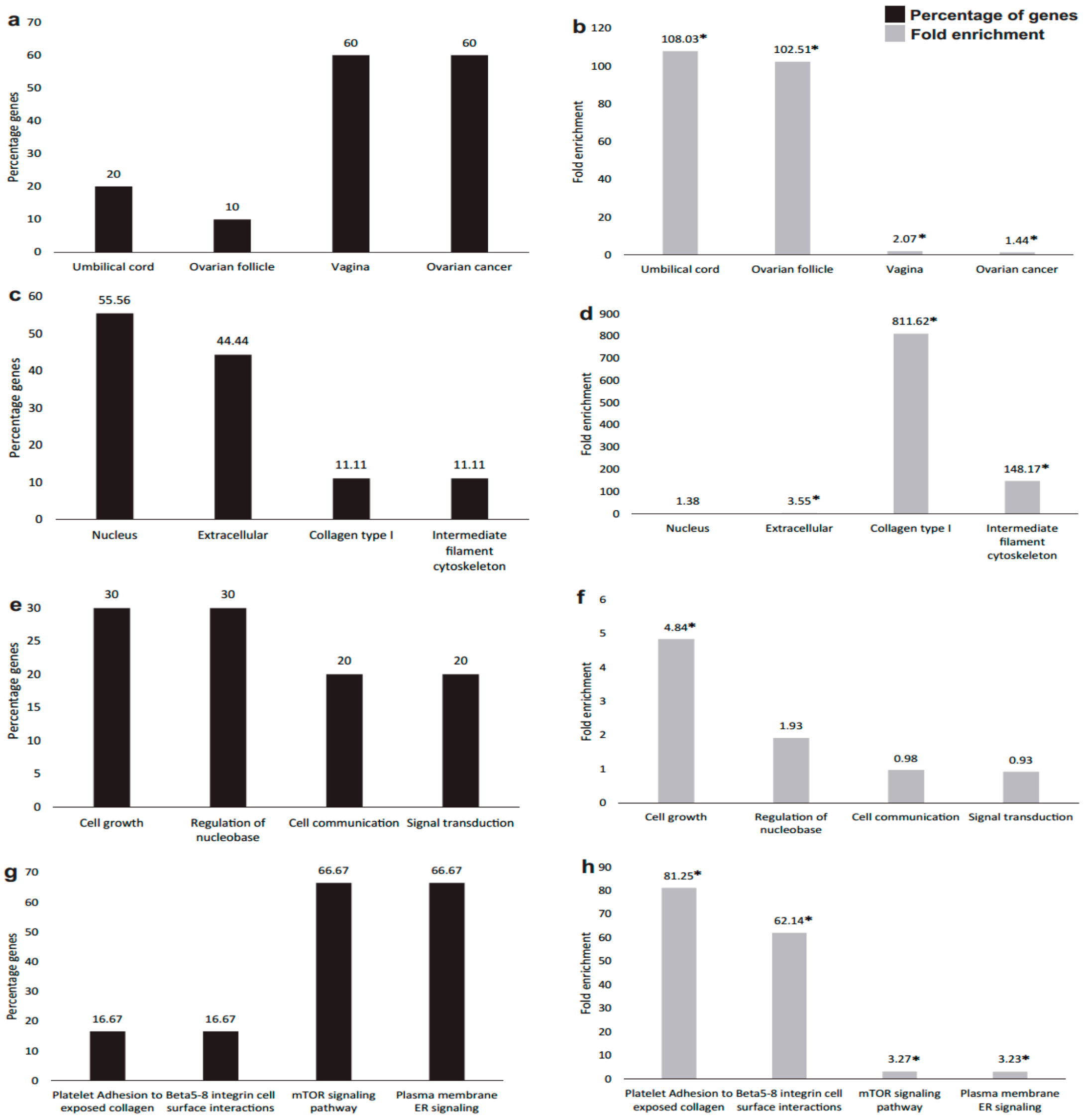

Figure S1. The functional enrichment in gene ontology terms in 14 genes in relation to site of expression (a,b), cellular components (c,d), biological processes (e,f) and biological pathways (g,h). *p-val<0.05.

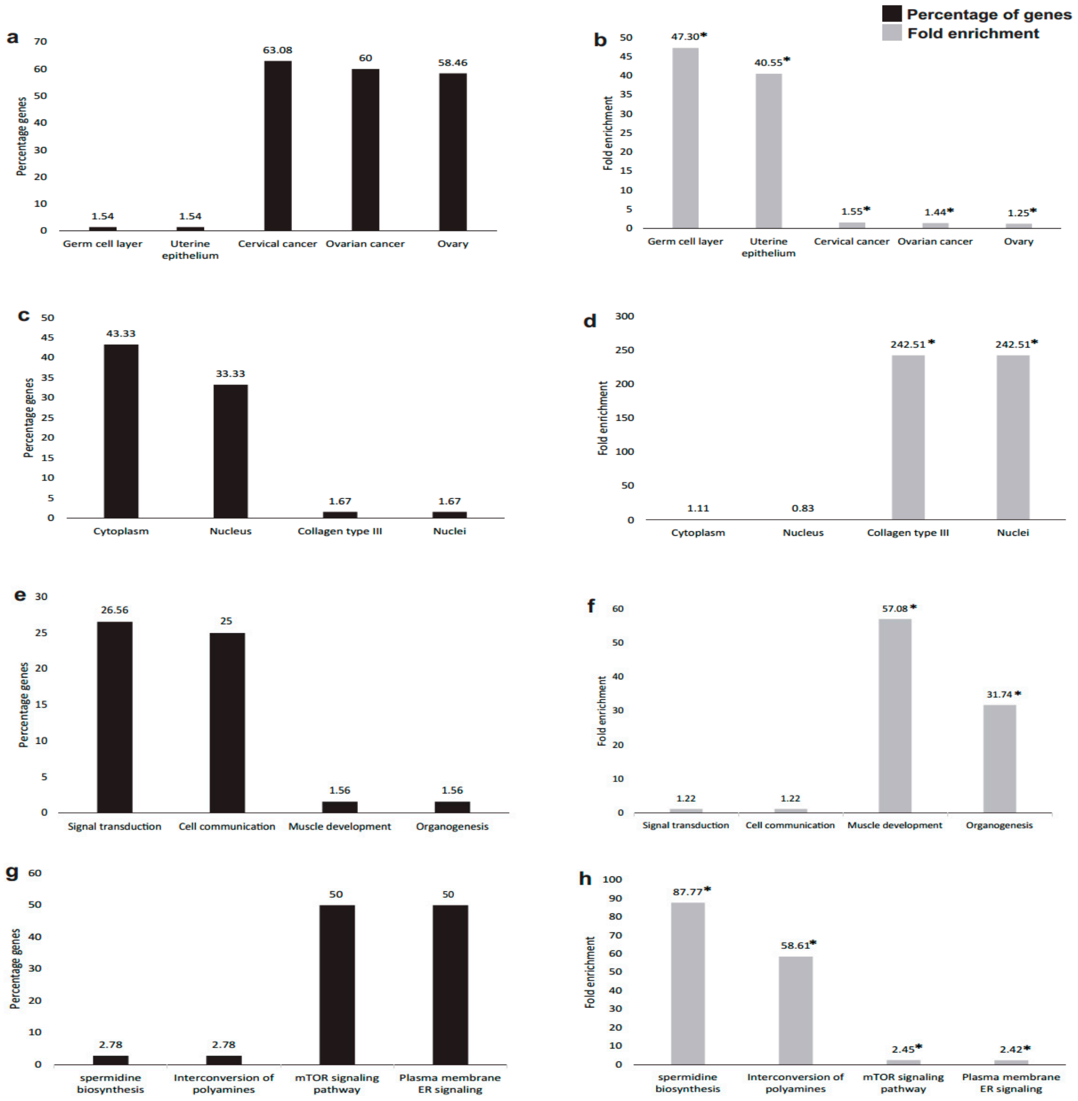

Figure S2. The functional enrichment in gene ontology terms in 80 genes in relation to site of expression (a,b), cellular components (c,d), biological processes (e,f) and biological pathways (g,h). *p-val<0.05.

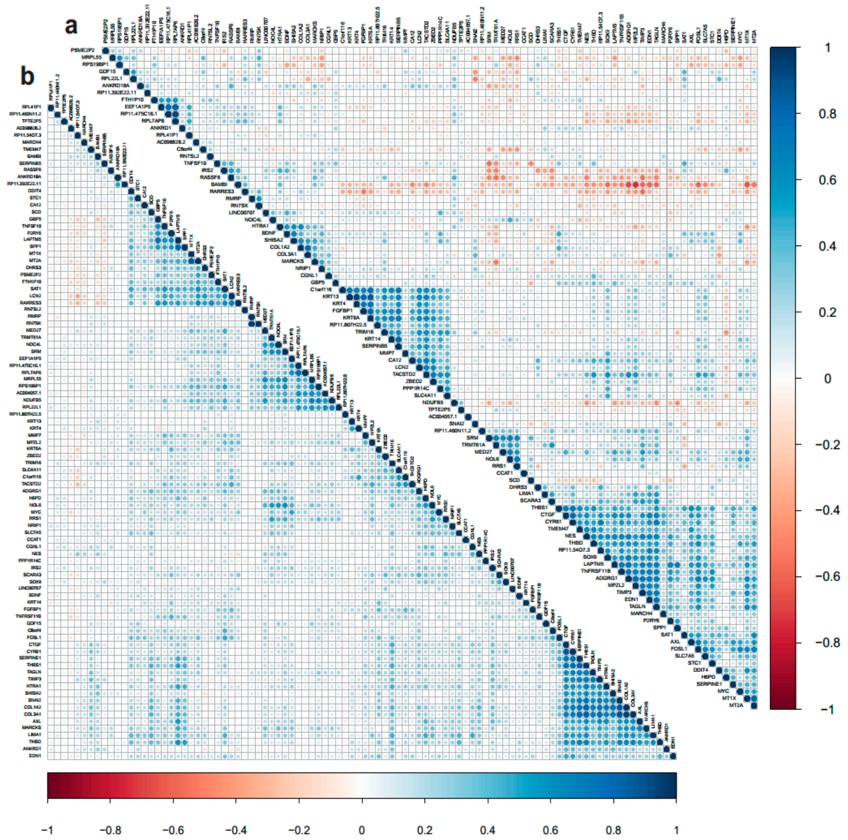

Figure S3. Heatmap of 94 genes in (a) normal ovarian tissue and (b) tumorous ovarian tissue showing correlation between these genes. Deep dark blue colour shows a strong correlation, while deep red colour shows no correlation.

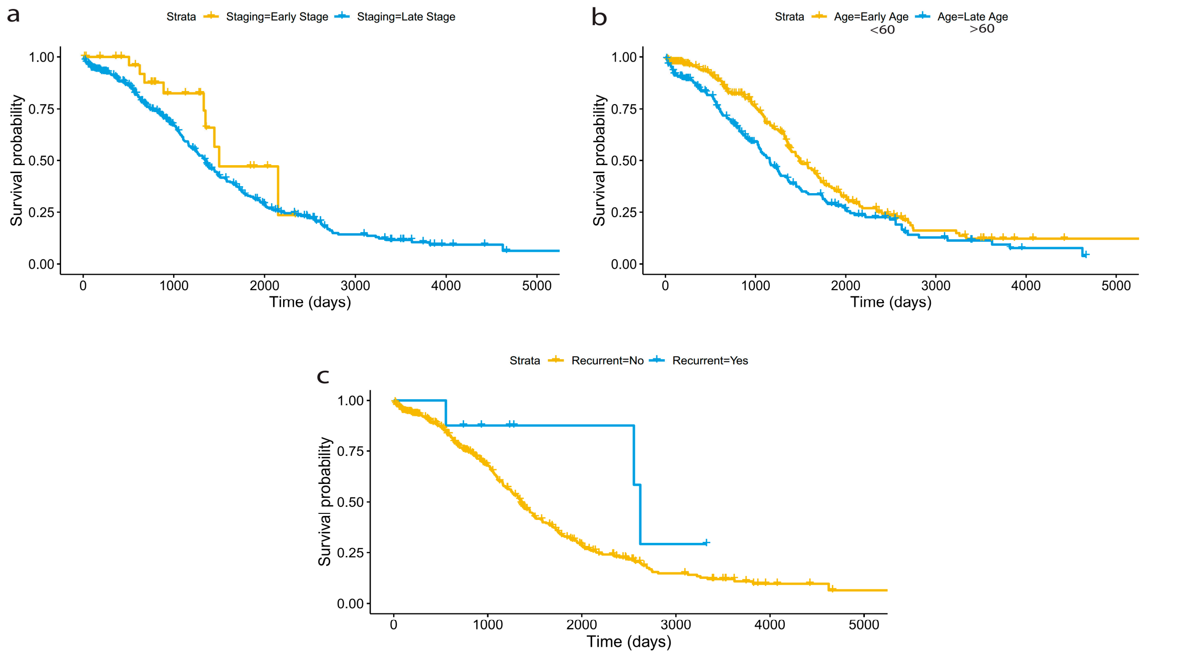

Figure S4. KM-plots for stratifying by (a) stage (late – III& IV vs early – I&II), (b) age (late – >60 vs early – <60), and (c) recurrent disease (yes vs no).

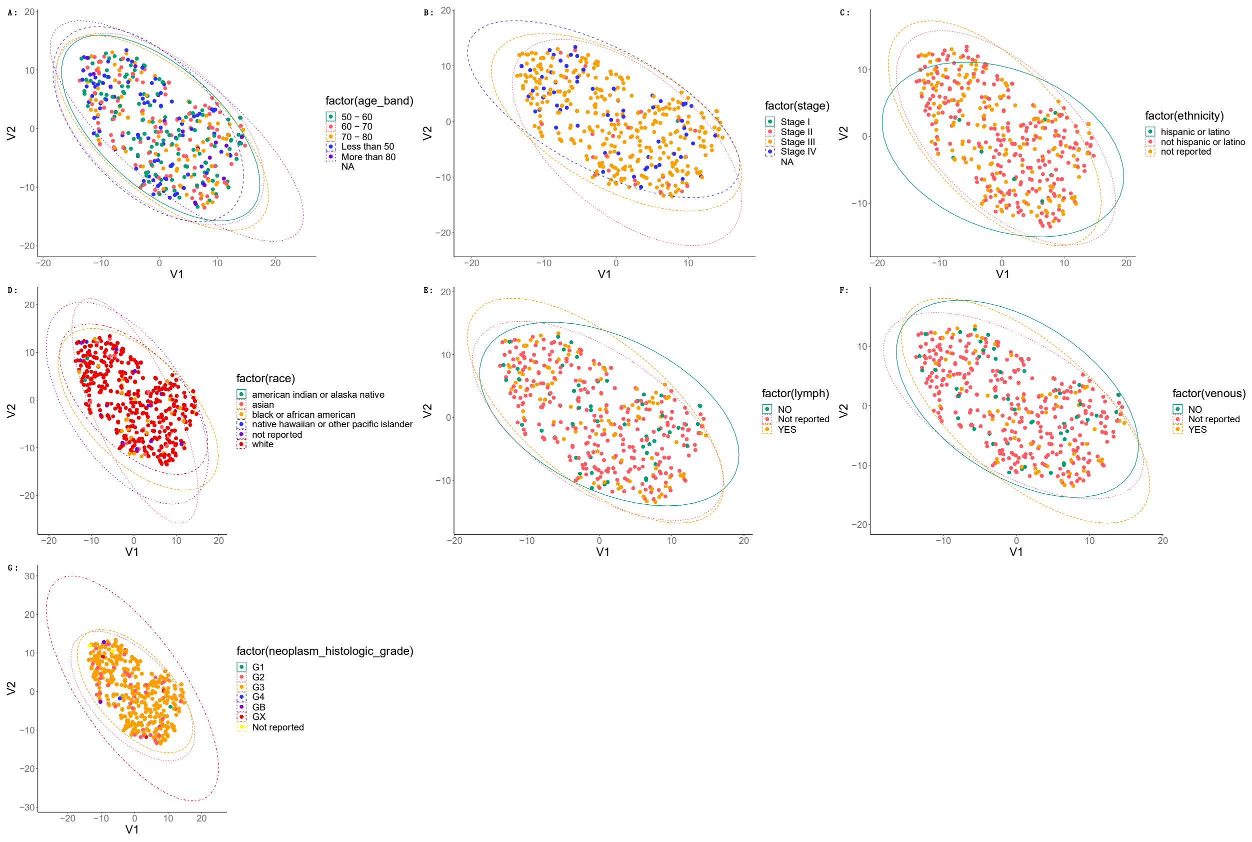

Figure S5. tSNE discrimination between various phenotypes using the information from the 94 gene expression profiles.

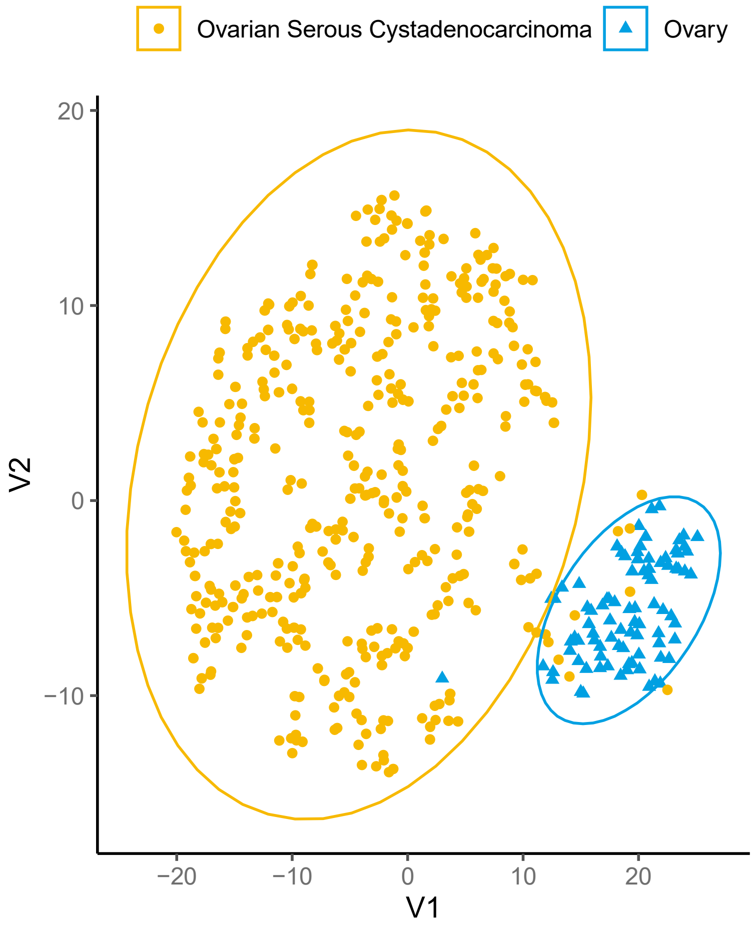

Figure S6. tSNE discrimination between tumour and normal samples using the information from the GBP5, SCL4A11 and LINC0070 gene expression profiles.

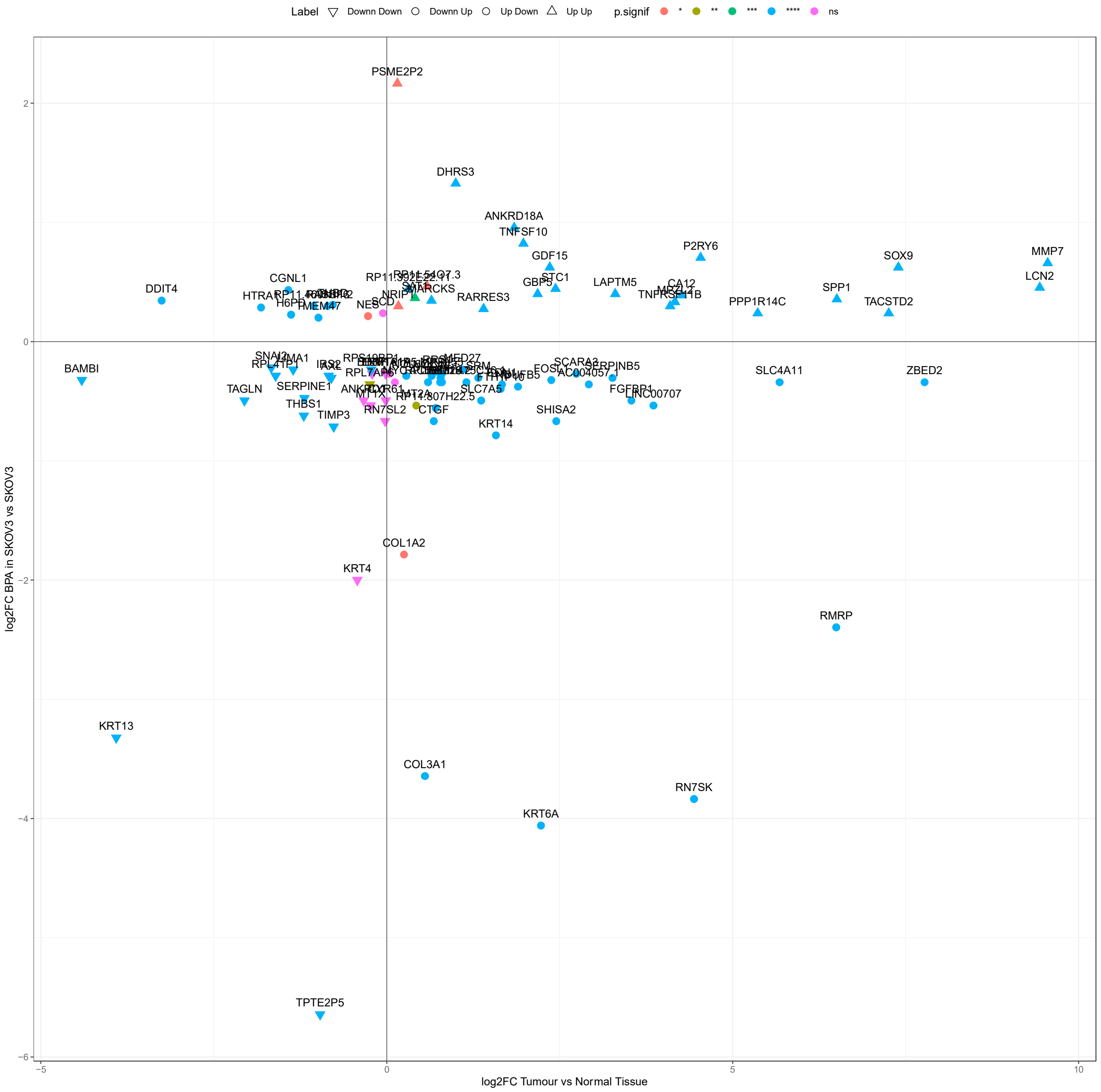

Figure S7. Scatter plot of the expression changes upon BPA exposure as compared to the changes in expression driven by ovarian cancer alone. The labels indicate the pairing in the change in expression in cancer as in SKOV3 cell lines under BPA treatment as compared to their respective controls. The colours are indicative of the statistical significance of the change in expression in ovarian tumour samples vs normal healthy tissue.

Table S1. Details of the clinicopathological features of the tissues used for the microarray.

#	Tissue type	STAGES
A1	Clear cell carcinoma	I
A2	Clear cell carcinoma	I
A3	Clear cell carcinoma	I
A4	Clear cell carcinoma	II
A5	Clear cell carcinoma (necrosis)	IIA
A6	Low grade serous carcinoma	IC
A7	Low grade serous carcinoma	IA
A8	Endometrioid adenocarcinoma	I
A9	Low grade serous carcinoma	IA
A10	Low grade serous carcinoma	IA
B1	Low grade serous carcinoma	I
B2	Low grade serous carcinoma	IA
B3	Low grade serous carcinoma	IB
B4	Low grade serous carcinoma	II
B5	High grade serous carcinoma	IIB
B6	High grade serous carcinoma	I
B7	High grade serous carcinoma	I
B8	High grade serous carcinoma	I
B9	High grade serous carcinoma	I
B10	High grade serous carcinoma	IA
C1	High grade serous carcinoma	IIB
C2	High grade serous carcinoma	I
C3	High grade serous carcinoma	III
C4	High grade serous carcinoma	I
C5	High grade serous carcinoma	IA
C6	High grade serous carcinoma	IV
C7	High grade serous carcinoma	IB
C8	High grade serous carcinoma	IIIC
C9	High grade serous carcinoma	I
C10	High grade serous carcinoma	IIIC
D1	High grade serous carcinoma	IA
D2	High grade serous carcinoma	IIIC
D3	High grade serous carcinoma	IC
D4	High grade serous carcinoma	IIIC
D5	High grade serous carcinoma	IA
D6	High grade serous carcinoma	I
D7	High grade serous carcinoma	IA
D8	High grade serous carcinoma	I
D9	High grade serous carcinoma	IA
D10	High grade serous carcinoma	I

E1	High grade serous carcinoma	IIIC
E2	High grade serous carcinoma	I
E3	High grade serous carcinoma with necrosis	II
E4	High grade serous carcinoma	IIIC
E5	High grade serous carcinoma	IC
E6	High grade serous carcinoma	IIIC
E7	High grade serous carcinoma	II
E8	High grade serous carcinoma	II
E9	High grade serous carcinoma	II
E10	High grade serous carcinoma	I
F1	High grade serous carcinoma with necrosis	IC
F2	High grade serous carcinoma (sparse)	IA
F3	High grade serous carcinoma	II
F4	High grade serous carcinoma	IIIC
F5	High grade serous carcinoma	IA
F6	High grade serous carcinoma with necrosis	IC
F7	High grade serous carcinoma	IIIC
F8	High grade serous carcinoma	IIIC
F9	High grade serous carcinoma	IIIC
F10	High grade serous carcinoma	IIIC
G1	High grade serous carcinoma	II
G2	High grade serous carcinoma	IA
G3	High grade serous carcinoma	III
G4	High grade serous carcinoma	I
G5	High grade serous carcinoma	IIIA
G6	High grade serous carcinoma	IIB
G7	High grade serous carcinoma	IA
G8	High grade serous carcinoma	IA
G9	Mucinous papillary adenocarcinoma (necrosis)	I
G10	Endometrioid adenocarcinoma	II
H1	Mucinous adenocarcinoma	IB
H2	Mucinous adenocarcinoma	IA
H3	Mucinous adenocarcinoma with necrosis	IIA
H4	Mucinous adenocarcinoma	IB
H5	Mucinous adenocarcinoma with necrosis	IIIC
H6	Mucinous adenocarcinoma	IB
H7	Mucinous adenocarcinoma	I
H8	Mucinous adenocarcinoma	III
H9	Mucinous adenocarcinoma	IA
H10	Endometrioid adenocarcinoma	IA
I1	Metastatic serous carcinoma from ovary	-
I2	Metastatic serous carcinoma from ovary	-

I3	Metastatic serous carcinoma from ovary	-
I4	Metastatic serous carcinoma from ovary	-
I5	Metastatic serous carcinoma from ovary	-
I6	Metastatic clear cell carcinoma from ovary	-
I7	Metastatic serous carcinoma of fibrofatty tissue from ovary of No.64	-
I8	Metastatic serous carcinoma from ovary	-
I9	Metastatic serous carcinoma from ovary	-
I10	Metastatic serous carcinoma of fibrofatty tissue from ovary	-
J1	Adjacent normal ovary tissue	-
J2	Adjacent normal ovary tissue	-
J3	Adjacent normal ovary tissue	-
J4	Adjacent normal ovary tissue	-
J5	Adjacent normal ovary tissue	-
J6	Adjacent normal ovary tissue	-
J7	Adjacent normal ovary tissue	-
J8	Adjacent normal ovary tissue	-
J9	Adjacent normal ovary tissue	-
J10	Adjacent normal ovary tissue	-

Table S2. List of genes associated with the phenotypes in **Figure 2**.

Phenotypes	Genes
Site of Expression (14 genes)	
Umbilical cord	*MT1X; NES*
Ovarian follicle	*BDNF*
Vagina	*MYC; SCD; CYR61; BDNF; KRT4; NES*
Ovarian cancer	*MYC; SCD; CYR61; BDNF; KRT4; NES*
Cellular Components (14 genes)	
Nucleus	*MYC; MT1X; SCD; ANKRD1; NES*
Extracellular	*MT1X; COL1A2; CYR61; BDNF*
Collagen type I	*COL1A2*
Intermediate filament cytoskeleton	*NES*
Biological Processes (14 genes)	
Cell growth	*COL1A2; KRT4; NES*
Regulation of nucleobase	*MYC; TRMT61A; ANKRD1*
Cell communication	*CYR61; BDNF*
Signal transduction	*CYR61; BDNF*
Biological Pathways (14 genes)	
Platelet Adhesion to exposed collagen	*COL1A2*
Beta5-8 integrin cell surface interactions	*CYR61*
mTOR signaling pathway	*MYC; COL1A2; CYR61; BDNF*
Plasma membrane ER signaling	*MYC; COL1A2; CYR61; BDNF*

Site of Expression (80 genes)	
Germ cell layer	*HTRA1*
Uterine epithelium	*CA12*
Cervical cancer	*TNFRSF11B; NDUFB5; RARRES3; MMP7; LCN2; SOX9; C1orf116; SPP1; P2RY6; SRM; SLC4A11; CA12; FGFBP1; SERPINB5; STC1; FOSL1; GDF15; GBP5; MED27; MRPL55; MARCKS; BAMBI; KRT13; DDIT4; SNAI2; CGNL1; LIMA1; KRT14; DHRS3; TRIM16; CTGF; COL3A1; TIMP3; THBD; IRS2; C8orf4; SERPINE1; H6PD; TAGLN; EDN1; ZBED2*
Ovarian cancer	*TNFRSF11B; NDUFB5; RARRES3; MMP7; LCN2; SOX9; C1orf116; SPP1; P2RY6; SRM; SLC4A11; CA12; FGFBP1; SERPINB5; STC1; FOSL1; GDF15; GBP5; TNFSF10; EDN1; MED27; MRPL55; MARCKS; BAMBI; DDIT4; SNAI2; LIMA1; KRT14; DHRS3; TRIM16; CTGF; COL3A1; TIMP3; IRS2; C8orf4; SERPINE1; H6PD; TAGLN ; ZBED2*
Ovary	*TNFRSF11B; NDUFB5; RARRES3; MMP7; LCN2; TACSTD2; C1orf116; SPP1; P2RY6; SRM; SLC4A11; CA12; SERPINB5; STC1; FOSL1; GDF15; GBP5;; MRPL55; MARCKS; KRT13; DDIT4; HTRA1; SNAI2; LIMA1; THBS1; KRT6A; KRT14; DHRS3; TRIM16; COL3A1; NOL6; TIMP3; IRS2; C8orf4; H6PD; TAGLN; MED27; CTGF*
**Cellular Components (80 genes)**	
Cytoplasm	*TACSTD2; C1orf116; SRM; PPP1R14C; FGFBP1; SERPINB5; SCARA3; STC1; EDN1; MED27; MARCKS; SAT1; BAMBI; KRT13; DDIT4; LIMA1; THBS1; KRT6A; KRT14; TRIM16; MT2A; NRIP1; IRS2; SERPINE1; H6PD; TAGLN*
Nucleus	*RARRES3; ZBED2; SOX9; STC1; FOSL1; TNFSF10; MED27; SAT1; KRT13; DDIT4; SNAI2; LIMA1; THBS1; KRT14; TRIM16; RRS1; MT2A; NOL6; NRIP1; IRS2*
Collagen type III	*COL3A1*
Nuclei	*MT2A*
**Biological Processes (80 genes)**	
Signal transduction	*TNFRSF11B; RARRES3; TACSTD2; P2RY6; ADGRG1; LAPTM5; PPP1R14C; FGFBP1; SCARA3; STC1; GDF15; GBP5; TNFSF10; EDN1; BAMBI; AXL; IRS2*
Cell communication	*TNFRSF11B; RARRES3; TACSTD2; P2RY6; LAPTM5; PPP1R14C; FGFBP1; SCARA3; STC1; GDF15; GBP5; TNFSF10; EDN1; BAMBI; AXL; IRS2*
Muscle development	*TAGLN*

Organogenesis	*MPZL2*
**Biological Pathways (80 genes)**	
spermidine biosynthesis	*SRM*
Interconversion of polyamines	*SAT1*
mTOR signaling pathway	*MMP7; SPP1; SERPINB5; FOSL1; GDF15; EDN1; BAMBI; DDIT4; SNAI2; LIMA1; KRT14; CTGF; MT2A; NRIP1; THBD; IRS2; SERPINE1; TAGLN*
Plasma membrane ER signaling	*MMP7; SPP1; SERPINB5; FOSL1; GDF15; EDN1; BAMBI; DDIT4; SNAI2; LIMA1; KRT14; CTGF; MT2A; NRIP1; THBD; IRS2; SERPINE1; TAGLN*

Table S3. Gene set enrichment analysis results for 94 BPA dysregulated genes.

GENE	Rank	Test	Res	Core enrichment
SRM	57914	-28.8	0.0114	YES
MARCH4	57125	-22.3	-0.0165	YES
BAMBI	56716	-20.5	-0.0415	YES
LINC00707	56537	-19.8	-0.0679	YES
P2RY6	56320	-19	-0.0926	YES
FGFBP1	56288	-18.9	-0.119	YES
PPP1R14C	54609	-15.3	-0.118	YES
ZBED2	54590	-15.3	-0.139	YES
FTH1P10	54514	-15.2	-0.16	YES
MARCKS	54288	-14.8	-0.178	YES
FOSL1	54141	-14.6	-0.197	YES
SERPINB5	52835	-12.9	-0.195	YES
TNFRSF11B	52587	-12.6	-0.21	YES
CGNL1	52335	-12.3	-0.223	YES
TMEM47	52005	-12	-0.235	YES
SHISA2	52000	-12	-0.253	YES
RPS19BP1	51190	-11.2	-0.256	YES
SAT1	50681	-10.7	-0.263	YES
PSME2P2	49970	-10.1	-0.266	YES
C1orf116	49696	-9.86	-0.276	YES
MMP7	49468	-9.69	-0.287	YES
EDN1	49133	-9.45	-0.295	YES
AC098828.2	49087	-9.42	-0.308	YES
ANKRD18A	48242	-8.88	-0.307	YES
MYC	47592	-8.46	-0.308	YES
CCAT1	47590	-8.46	-0.32	YES
COL1A2	47051	-8.12	-0.323	YES

RP11-807H22.5	46789	-7.94	-0.331	YES
RMRP	46472	-7.72	-0.337	YES
BDNF	46272	-7.61	-0.344	YES
COL3A1	45870	-7.37	-0.348	YES
NDUFB5	45825	-7.34	-0.358	YES
RP11-54O7.3	44782	-6.73	-0.351	YES
AXL	44343	-6.49	-0.353	YES
SERPINE1	43816	-6.21	-0.353	YES
EEF1A1P5	43789	-6.2	-0.362	YES
SNAI2	43737	-6.17	-0.37	YES
SCD	43577	-6.08	-0.376	YES
GDF15	43530	-6.05	-0.384	YES
CTGF	43302	-5.94	-0.389	YES
THBD	43226	-5.9	-0.396	YES
RP11-392E22.11	43093	-5.84	-0.402	YES
RASSF6	42928	-5.76	-0.408	YES
NOL6	42355	-5.48	-0.406	YES
GBP5	42043	-5.34	-0.409	YES
LIMA1	41545	-5.1	-0.408	YES
KRT14	41076	-4.89	-0.407	YES
C8orf4	40959	-4.84	-0.412	YES
CA12	40417	-4.62	-0.41	YES
MT2A	40189	-4.51	-0.413	YES
IRS2	39985	-4.42	-0.416	YES
RPL7AP6	39979	-4.41	-0.422	YES
DHRS3	38626	-3.9	-0.405	NO
STC1	38617	-3.9	-0.411	NO
KRT6A	38074	-3.7	-0.407	NO
NRIP1	37754	-3.58	-0.407	NO
MT1X	37072	-3.34	-0.4	NO
TIMP3	36940	-3.3	-0.403	NO
CYR61	36407	-3.15	-0.399	NO
LCN2	36049	-3.04	-0.397	NO
TRIM16	34201	-2.55	-0.37	NO
ANKRD1	34126	-2.54	-0.372	NO
NES	32473	-2.18	-0.348	NO
KRT4	31712	-2.02	-0.338	NO
TPTE2P5	29299	-1.63	-0.299	NO
TRMT61A	26245	-1.21	-0.25	NO
AC004057.1	26108	-1.19	-0.249	NO
THBS1	26012	-1.17	-0.249	NO
TAGLN	21281	-0.334	-0.17	NO
RP11-475C16.1	20450	-0.0528	-0.156	NO

SLC4A11	11635	0.516	-0.00559	NO
RPL22L1	11041	0.69	0.00381	NO
RN7SL2	9215	1.14	0.034	NO
TNFSF10	7879	1.68	0.0552	NO
SCARA3	6547	2.48	0.0756	NO
HTRA1	6181	2.74	0.0782	NO
DDIT4	6036	2.88	0.0768	NO
H6PD	5620	3.29	0.0797	NO
RARRES3	4970	3.98	0.0861	NO
RP11-460N11.2	4690	4.31	0.0851	NO
SLC7A5	4194	4.93	0.0874	NO
SOX9	4073	5.13	0.0824	NO
LAPTM5	3889	5.41	0.0782	NO
MED27	3720	5.71	0.0733	NO
SPP1	3495	6.07	0.0689	NO
RRS1	2615	7.9	0.0752	NO
MRPL55	2537	8.03	0.0652	NO
NOC4L	2531	8.04	0.0537	NO
RPL41P1	2378	8.42	0.0448	NO
KRT13	2366	8.45	0.0329	NO
ADGRG1	2255	8.79	0.0226	NO
TACSTD2	2048	9.43	0.0135	NO
RN7SK	1989	9.61	0.000983	NO
MPZL2	964	14.7	0.00469	NO

Table S4. Gene expression rank in Hui et al, TCGA, and GTEx datasets.

Gene Name	Gene ID	TCGA	GTEx	SKOV3 w BPA	SKOV3
AC004057.1	ENSG00000196656	3	16	70	74
AC098828.2	ENSG00000234378	76	77	76	75
ANKRD1	ENSG00000148677	75	64	64	67
ANKRD18A	ENSG00000180071	73	71	57	59
AXL	ENSG00000167601	33	19	2	2
BAMBI	ENSG00000095739	64	20	54	53
BDNF	ENSG00000176697	71	59	38	35
CA12	ENSG00000074410	45	60	19	24
CGNL1	ENSG00000128849	50	30	20	30
COL1A2	ENSG00000164692	1	1	71	66
COL3A1	ENSG00000168542	2	4	75	64
DDIT4	ENSG00000168209	25	3	32	42
DHRS3	ENSG00000162496	24	23	62	73

EDN1	ENSG00000078401	60	51	45	40
EEF1A1P5	ENSG00000196205	12	10	27	22
FGFBP1	ENSG00000137440	70	74	7	5
FOSL1	ENSG00000175592	63	61	9	9
FTH1P10	ENSG00000223361	62	58	74	76
GBP5	ENSG00000154451	52	52	47	51
GDF15	ENSG00000130513	51	53	56	61
H6PD	ENSG00000049239	18	7	17	21
HTRA1	ENSG00000166033	22	5	24	27
IRS2	ENSG00000185950	29	14	50	47
KRT13	ENSG00000171401	74	45	78	70
KRT14	ENSG00000186847	61	57	65	65
KRT4	ENSG00000170477	65	49	69	60
KRT6A	ENSG00000205420	53	54	77	71
LAPTM5	ENSG00000162511	10	40	60	68
LCN2	ENSG00000148346	9	68	44	50
LIMA1	ENSG00000050405	35	15	22	14
LINC00707	ENSG00000238266	72	76	63	62
MARCKS	ENSG00000277443	19	18	13	19
MED27	ENSG00000160563	43	42	43	41
MMP7	ENSG00000137673	23	73	6	7
MPZL2	ENSG00000149573	27	47	28	34
MRPL55	ENSG00000162910	31	36	48	46
MT1X	ENSG00000187193	40	26	55	52
MT2A	ENSG00000125148	11	13	3	3
MYC	ENSG00000136997	16	11	21	12
NDUFB5	ENSG00000136521	17	28	18	17
NES	ENSG00000132688	42	29	26	25
NOC4L	ENSG00000184967	39	37	41	36
NOL6	ENSG00000165271	28	22	8	8
NRIP1	ENSG00000180530	32	27	12	16
P2RY6	ENSG00000171631	55	66	58	63
PPP1R14C	ENSG00000198729	58	69	39	44
PSME2P2	ENSG00000225131	66	56	73	78
RASSF6	ENSG00000169435	69	50	29	37
RN7SL2	ENSG00000274012	78	78	67	58
RPL22L1	ENSG00000163584	34	35	51	49
RPL41P1	ENSG00000227063	77	72	72	77
RPL7AP6	ENSG00000242071	56	43	59	57
RPS19BP1	ENSG00000187051	36	24	31	28
RRS1	ENSG00000179041	37	39	34	32
SAT1	ENSG00000130066	6	9	40	45

SCARA3	ENSG00000168077	8	31	25	15
SCD	ENSG00000099194	20	12	11	13
SERPINB5	ENSG00000206075	67	70	30	23
SERPINE1	ENSG00000106366	38	17	46	39
SHISA2	ENSG00000180730	68	67	61	56
SLC4A11	ENSG00000088836	41	62	33	31
SLC7A5	ENSG00000103257	30	38	1	1
SNAI2	ENSG00000019549	57	33	42	38
SOX9	ENSG00000125398	26	65	66	72
SPP1	ENSG00000118785	4	46	5	6
SRM	ENSG00000116649	15	21	16	11
STC1	ENSG00000159167	49	48	53	54
TACSTD2	ENSG00000184292	5	55	14	20
TAGLN	ENSG00000149591	7	2	36	29
THBD	ENSG00000178726	54	41	23	26
THBS1	ENSG00000137801	14	6	4	4
TIMP3	ENSG00000100234	13	8	68	69
TMEM47	ENSG00000147027	46	25	15	18
TNFRSF11B	ENSG00000164761	59	63	35	43
TNFSF10	ENSG00000121858	21	32	49	55
TRIM16	ENSG00000221926	48	44	52	48
TRMT61A	ENSG00000166166	44	34	37	33
ZBED2	ENSG00000177494	47	75	10	10
